# Asymmetric distribution of phosphatidylserine is generated in the absence of phospholipid flippases in *Saccharomyces cerevisiae*

**DOI:** 10.1002/mbo3.211

**Published:** 2014-09-13

**Authors:** Tetsuo Mioka, Konomi Fujimura-Kamada, Kazuma Tanaka

**Affiliations:** 1 Division of Molecular Interaction, Institute for Genetic Medicine, Hokkaido University Graduate School of Life ScienceN15 W7, Kita-ku, Sapporo, 060-0815, Japan

**Keywords:** Flippase, membrane traffic, phosphatidylserine, phospholipid asymmetry, secretory pathway, yeast

## Abstract

In eukaryotic cells, phosphatidylserine (PS) is predominantly located in the cytosolic leaflet of the plasma membrane; this asymmetry is generated by an unknown mechanism. In this study, we used the PS-specific probe mRFP-Lact-C2 to investigate the possible involvement of type 4 P-type ATPases, also called phospholipid flippases, in the generation of this asymmetry in *Saccharomyces cerevisiae*. PS was not found in the *trans*-Golgi Network in wild-type cells, but it became exposed when vesicle formation was compromised in the *sec7* mutant, and it was also exposed on secretory vesicles (SVs), as reported previously. However, flippase mutations did not reduce the exposure of PS in either case, even at low levels that would only be detectable by quantitative analysis of mRFP-Lact-C2 fluorescence in isolated SVs. Furthermore, no reduction in the PS level was observed in a mutant with multiple flippase mutations. Because PS was not exposed in a mutant that accumulates ER or *cis*/medial-Golgi membranes, Golgi maturation seems to be a prerequisite for PS translocation. Our results suggest that an unknown mechanism, possibly a protein with flippase-like activity, acts in conjunction with known flippases to regulate PS translocation.

## Introduction

Phospholipids in the eukaryotic plasma membrane are unevenly distributed between the cytosolic and exoplasmic leaflets. Phosphatidylcholine (PC) is mainly located in the exoplasmic leaflet, whereas phosphatidylserine (PS) and phosphatidylethanolamine (PE) are mainly located in the cytosolic leaflet (Daleke 2003). In apoptotic cells, this phospholipid asymmetry is disrupted, and exposure of PS to the exoplasmic leaflet is recognized as an “eat me” signal (Ravichandran and Lorenz 2007). Changes in phospholipid asymmetry are also involved in the regulation of cell polarity through the control of Cdc42p and its associated factors (Saito et al. 2007; Fairn et al. 2011a; Das et al. 2012). On the other hand, less is known about phospholipid asymmetry in endomembrane organelles. The GFP-tagged C2 domain of lactadherin (GFP-Lact-C2), a milk glycoprotein, specifically binds to PS in a calcium-independent manner, and thus enables the visualization of endogenous PS in the cytosolic leaflet of membranes (Yeung et al. 2008). In previous studies, GFP-Lact-C2 revealed that PS distribution in mammalian cells is uneven: it is present on the cytosolic faces of the plasma membrane, endocytic organelles, and the *trans*-Golgi Network (TGN), but not that of the endoplasmic reticulum (ER); instead, PS appears to be present in the luminal leaflet in the ER and early Golgi (Yeung et al. 2008; Fairn et al. 2011b; Kay et al. 2012). In yeast, GFP-Lact-C2 is exclusively localized to the plasma membrane in wild-type cells, and is also present on secretory vesicles (SVs) in secretory pathway mutants (Fairn et al. 2011a). These results suggest that translocation of PS from the luminal to cytosolic leaflet occurs during exit from the TGN in the secretory pathway, thereby generating PS asymmetry in the plasma membrane. Because PS is a negatively charged lipid, changes in the content of PS along the secretory pathway generate a gradient of surface charge, which seems to govern proper localization of peripheral proteins (Yeung et al. 2008; Bigay and Antonny 2012; Cho et al. 2012).

Phospholipid flippases are candidate factors responsible for generating PS asymmetry in the plasma membrane. Flippases are type 4 P-type ATPases that are believed to translocate phospholipids from the exoplasmic to cytosolic leaflet (flip) (Daleke 2007; Lenoir et al. 2007; Tanaka et al. 2011; Sebastian et al. 2012). In budding yeast, there are five flippases: Drs2p, Dnf1p, Dnf2p, Dnf3p, and Neo1p. Drs2p, Dnf1/2p, and Dnf3p form complexes with Cdc50p, Lem3p, and Crf1p noncatalytic subunits, respectively, and these interactions are required for ER exit, proper localization, function, and activity of the flippases (Saito et al. 2004; Furuta et al. 2007; Lenoir et al. 2009; Takahashi et al. 2011; Puts et al. 2012). Therefore, defects in *drs2*Δ, *dnf1*Δ *dnf2*Δ, and *dnf3*Δ mutants are phenocopied by *cdc50*Δ, *lem3*Δ, and *crf1*Δ mutants, respectively (Saito et al. 2004; Furuta et al. 2007).

Cdc50p–Drs2p resides primarily in the TGN and endosomes (Chen et al. 1999; Saito et al. 2004), and its ATP-dependent PS flippase activity has been demonstrated in isolated Golgi membranes (Natarajan et al. 2004), isolated SVs (Alder-Baerens et al. 2006), and an in vitro reconstitution system (Zhou and Graham 2009). Cdc50p–Drs2p has been implicated in the formation of clathrin-coated vesicles from the TGN (Gall et al. 2002; Liu et al. 2008), and work by our group showed that Cdc50p–Drs2p is involved in the endocytic recycling pathway, in which endocytosed proteins are transported to the TGN *via* early endosomes to be recycled back to the plasma membrane, in conjunction with Lem3p–Dnf1/2p and Crf1p–Dnf3p (Furuta et al. 2007). In the absence of these flippases, the Snc1p v-SNARE accumulates in enlarged early endosome-derived membranes due to defects in vesicle formation. We recently showed that Drs2p physically interacts with the F-box protein Rcy1p, which is specifically required for the endocytic recycling pathway (Hanamatsu et al. 2014).

Lem3p–Dnf1/2p is mainly localized to the plasma membrane (Kato et al. 2002; Pomorski et al. 2003), but like Cdc50p–Drs2p, this complex is also processed through the endocytic recycling pathway via early endosomes and the TGN (Saito et al. 2004; Liu et al. 2007). PS translocation by Lem3p–Dnf1/2p has been implicated in the sorting of Tat2p tryptophan transporter at the TGN (Hachiro et al. 2013). Crf1p–Dnf3p is localized to early endosome/TGN and plays a redundant role with Cdc50p–Drs2p and Lem3p–Dnf1/2p in growth and endocytic recycling; consistent with this, the *crf1*Δ and *dnf3*Δ single mutants do not exhibit a discernible phenotype (Hua et al. 2002; Pomorski et al. 2003; Furuta et al. 2007). Neo1p is different from other flippases in that it does not associate with a Cdc50 family member (Saito et al. 2004) and is independently essential for viability (Hua et al. 2002). Neo1p is involved in membrane trafficking from the *cis*-Golgi to the ER (Hua and Graham 2003), as well as within the endosomal-Golgi system (Wicky et al. 2004). Although Neo1p has not been demonstrated to have flippase activity, it is clear that the function of Neo1p in the endocytic recycling pathway is redundant with that of Cdc50p–Drs2p (Takeda et al. 2014).

All five yeast flippases are mainly or partially localized to endosomal/TGN membranes, suggesting that they may be involved in PS flipping at the TGN, and thus involved in generation of the plasma membrane PS asymmetry. In this study, we examined the contribution of flippases to the development of PS asymmetry in yeast, using mRFP-tagged Lact-C2 (mRFP-Lact-C2) as a probe for endogenous PS. Our results suggest that PS translocation occurs at the TGN concomitantly with the formation of SVs, even in the absence of flippases.

## Experimental Procedures

### Media and genetic techniques

Unless otherwise specified, strains were grown in rich medium (YPDA: 1% yeast extract (BD Difco, Franklin Lakes, NJ), 2% bacto-peptone (BD Difco), 2% glucose (Wako Pure Chemical Industries Ltd., Osaka, Japan), and 0.01% adenine (Wako Pure Chemical Industries Ltd.). Strains carrying plasmids were selected in synthetic medium (SD) containing the required nutritional supplements (Rose et al. 1990). For induction of the *GAL1* promoter, 3% galactose (Sigma-Aldrich, St. Louis, MO, USA) and 0.2% sucrose (Wako Pure Chemical Industries Ltd.) were used as carbon sources instead of glucose (YPGA, SG-Leu, SG-Ura, and SG-Leu-Ura). When required, the medium was supplemented with 1 mmol/L ethanolamine (Sigma) to support growth of the *cho1*Δ mutants. Standard genetic manipulations of yeast were performed as described previously (Guthrie and Fink 1991). Yeast transformations were performed by the lithium acetate method (Elble 1992; Gietz and Woods 2002). *Escherichia coli* strains DH5*α* and XL1-Blue were used for construction and amplification of plasmids.

### Strains and plasmids

Yeast strains used in this study are listed in Table1. PCR-based procedures were used to construct gene deletions and gene fusions with the *GAL1* promoter and mRFP1 (Longtine et al. 1998). The *sec6-4* (ANS6-2D), *sec7-1* (SF821-8A), *sec12-4* (MBY10-11D), *sec21-1* (MBY6-4D), and *sec23-1* (MBY8-20C) mutants were kind gifts from Dr. Akihiko Nakano (The University of Tokyo). The *gea1-4 gea2*Δ mutant was a kind gift from Dr. Catherine L. Jackson (Institut Jacques Monod). The *sec6-4* and *sec7-1* mutations were introduced into the YEF473 genetic background by three serial backcrosses. The *myo2-12* mutant in the YEF473 genetic background was from our laboratory stock (Yamamoto et al. 2010). All constructs produced by the PCR-based procedure were verified by colony-PCR amplification to confirm the replacement occurred at the expected locus. The plasmids used in this study are listed in Table2. The GFP-tagged Lact-C2 plasmid (pRS416-GFP-Lact-C2) (Yeung et al. 2008) was purchased from Haematologic Technologies, Inc. (Essex Junction, VT). pRS416-mRFP1-Lact-C2 was constructed by subcloning the coding region of mRFP1 to the *Hin*dIII–*Bgl*II gap of pRS416-GFP-Lact-C2. The *Kpn*I–*Sac*I mRFP1-Lact-C2 fragment was transferred to the integrating vector pRS306 to yield pRS306-mRFP1-Lact-C2 (pKT2108). pRS306-mRFP1-Lact-C2-AAA (Lact-C2-W26A, W33A, F34A) (pKT2131) was constructed by subcloning the coding region of mRFP1 to the *Hin*dIII–*Bgl*II gap of pRS306-GFP-Lact-C2-AAA (pKT1995) (Takeda et al. 2014). The *URA3::mRFP1-Lact-C2* (YKT1843) and *URA3::mRFP1-Lact-C2-AAA* (YKT1918) strains were constructed by integrating linearized pKT2108 and pKT2131 into the *URA3* locus, respectively. Schemes detailing construction of plasmids and DNA sequences of nucleotide primers are available upon request.

**Table 1:** *Saccharomyces cerevisiae* strains used in this study

Strain^1^		Genotype	Reference or source
ANS6-2D	*MATα*	*sec6-4 ura3-52 leu2-3,112 trp1-289 his3/4*	Gift from Dr. Akihiko Nakano
SF821-8A	*MAT***a**	*sec7-1 ura3-52 leu2-3,112 trp1-289 his4-580*	Gift from Dr. Akihiko Nakano
MBY10-11D	*MAT***a**	*sec12-4 ura3-52 leu2-3,112 trp1-289 his3/4*	Gift from Dr. Akihiko Nakano
MBY6-4D	*MATα*	*sec21-1 ura3-52 leu2-3,112 trp1-289 his3/4*	Gift from Dr. Akihiko Nakano
MBY8-20C	*MATα*	*sec23-1 ura3-52 leu2-3,112 trp1-289 his3/4*	Gift from Dr. Akihiko Nakano
CJY062-10-2	*MATα*	*ura3-52 leu2-3,112 his3-*Δ*200 lys2-801 ade2-101 gea1-4 gea2*Δ*::HIS3*	Peyroche et al. (2001)
YEF473	*MAT***a**/*α*	*lys2-810/lys2-810 ura3-52/ura3-52 his3*Δ*-200/his3*Δ*-200 trp1*Δ*-63/trp1*Δ*-63 leu2*Δ*-1/leu2*Δ*-1*	Longtine et al. (1998)
YKT38	*MAT***a**	*lys2-801 ura3-52 his3*Δ*-200 trp1*Δ*-63 leu2*Δ*-1*	Misu et al. (2003)
YKT249	*MAT***a**	*cdc50*Δ*::HIS3MX6*	Misu et al. (2003)
YKT1678	*MAT***a**	*myo2-12::HIS3MX6*	Our stock
YKT1843	*MAT***a**	*URA3::mRFP1-Lact-C2*	This study
YKT1844	*MAT***a**	*sec6-4 URA3::mRFP1-Lact-C2*	This study
YKT1845	*MATα*	*cho1*Δ*::hphMX4 URA3::mRFP1-Lact-C2*	This study
YKT1846	*MATα*	*sec6-4 cho1*Δ*::hphMX4 URA3::mRFP1-Lact-C2*	This study
YKT1847	*MAT***a**	*lem3*Δ*::TRP1 crf1*Δ*::hphMX4 URA3::mRFP1-Lact-C2*	This study
YKT1848	*MAT***a**	*sec6-4 lem3*Δ*::TRP1 crf1*Δ*::hphMX4 URA3::mRFP1-Lact-C2*	This study
YKT1849	*MAT***a**	*cdc50*Δ*::HIS3MX6 URA3::mRFP1-Lact-C2*	This study
YKT1850	*MAT***a**	*sec6-4 cdc50*Δ*::HIS3MX6 URA3::mRFP1-Lact-C2*	This study
YKT1660	*MAT***a**	*KanMX6::P*_*GAL1*_*-3HA-NEO1*	Takeda et al. (2014)
YKT1894	*MATα*	*sec6-4 KanMX6::P*_*GAL1*_*-3HA-NEO1*	This study
YKT1851	*MAT***a**	*KanMX6::P*_*GAL1*_*-3HA-NEO1 URA3::mRFP1-Lact-C2*	This study
YKT1852	*MAT***a**	*sec6-4 KanMX6::P*_*GAL1*_*-3HA-NEO1 URA3::mRFP1-Lact-C2*	This study
YKT1103	*MAT***a**	*KanMX6::P*_*GAL1*_*-3HA-CDC50 lem3*Δ*::TRP1 crf1*Δ*::HphMX4*	This study
YKT1853	*MATα*	*HIS3MX6::P*_*GAL1*_*-3HA-CDC50 lem3*Δ*::TRP1 crf1*Δ*::HphMX4 URA3::mRFP1-Lact-C2*	This study
YKT1854	*MAT***a**	*sec6-4 HIS3MX6::P*_*GAL1*_*-3HA-CDC50 lem3*Δ*::TRP1 crf1*Δ*::HphMX4 URA3::mRFP1-Lact-C2*	This study
YKT1855	*MAT***a**	*sec6-4 HIS3MX6::P*_*GAL1*_*-3HA-CDC50 lem3*Δ*::TRP1 crf1*Δ*::HphMX4*	This study
YKT1856	*MAT***a**	*KanMX6::P*_*GAL1*_*-3HA-CDC50 KanMX6::P*_*GAL1*_*-3HA-NEO1 lem3*Δ*::HIS3MX6 crf1*Δ*::hphMX4*URA3::mRFP1-Lact-C2	This study
YKT1857	*MATα*	*sec7-1 URA3::mRFP1-Lact-C2*	This study
YKT1858	*MATα*	*sec7-1 cho1*Δ*::HphMX4 URA3::mRFP1-Lact-C2*	This study
YKT1859	*MAT***a**	*sec7-1 cdc50*Δ*::HIS3MX6 URA3::mRFP1-Lact-C2*	This study
YKT1860	*MATα*	*sec7-1 lem3*Δ*::TRP1 crf1*Δ*::hphMX4 URA3::mRFP1-Lact-C2*	This study
YKT1670	*MAT***a**	*SEC7-mRFP1::TRP1*	This study
YKT1149	*MAT***a**	*cdc50*Δ*::HIS3MX6 SEC7-mRFP1::TRP1*	This study
YKT1861	*MATα*	*KanMX6::P*_*GAL1*_*-3HA-NEO1 SEC7-mRFP1::TRP1*	This study
YKT1862	*MAT***a**	*HIS3MX6::P*_*GAL1*_*-3HA-CDC50 lem3*Δ*::TRP1 crf1*Δ*::HphMX4 SEC7-mRFP1::KanMX6*	This study
YKT1863	*MATα*	*TRP1::P*_*GAL1*_*-3HA-PIK1 URA3::mRFP1-Lact-C2*	This study
YKT1918	*MAT***a**	*URA3::mRFP1-Lact-C2-AAA*	This study
YKT1919	*MATα*	*sec6-4 URA3::mRFP1-Lact-C2-AAA*	This study


Only relevant genotypes are described.

1 YKT strains are isogenic derivatives of YEF473.

**Table 2:** Plasmids used in this study

Plasmid	Characteristics	Reference or source
pKT1444 [pRS416 GFP-SNC1 pm]	*P*_*TPI1*_*-GFP-SNC1 pm URA3 CEN*	Lewis et al. (2000)
pKT1491 [pRS315 GFP-SNC1 pm]	*P*_*TPI1*_*-GFP-SNC1 pm LEU2 CEN*	This study
pKT1563 [pRS416 mRFP1-SNC1]	*P*_*TPI1*_*-mRFP1-SNC1 URA3 CEN*	Furuta et al. (2007)
pKT1568 [pRS315 mRFP1-SNC1]	*P*_*TPI1*_*-mRFP1-SNC1 LEU2 CEN*	Takeda et al. (2014)
pKT1749 [pRS416 GFP-Lact-C2]	GFP-Lact-C2 URA3 CEN	Yeung et al. (2008)
pKT1755 [pRS416 mRFP1-Lact-C2]	*mRFP1-Lact-C2 URA3 CEN*	This study
pKT1995 [pRS306-GFP-Lact-C2-AAA]	*GFP-Lact-C2-AAA URA3*	Takeda et al. (2014)
pKT2108 [pRS306-mRFP1-Lact-C2]	*mRFP1-Lact-C2 URA3*	This study
pKT2131 [pRS306-mRFP1-Lact-C2-AAA]	*mRFP1-Lact-C2-AAA URA3*	This study

### Determination of mRFP-Lact-C2 fluorescence of isolated SVs

SVs were isolated using a previously described protocol (Harsay and Bretscher 1995) with minor modifications. Unless otherwise specified, chemicals and reagents were purchased from Wako Pure Chemical Industries Ltd. Briefly, cells were grown at 25 or 30°C to early to mid-logarithmic phase (OD_600_ of 0.5–0.7) in 0.5 L of YPDA or SD, followed by further incubation at 37°C for 2 h to allow accumulation of SVs. The cells (∼500 OD_600_ units) were then collected and converted to spheroplasts in spheroplast wash buffer (1.4 mol/L sorbitol [Sigma], 50 mmol/L KP_i_ at pH 7.4, 10 mmol/L sodium azide) containing 90 *μ*g mL^−1^ Zymolyase 100T (Seikagaku Corp., Tokyo, Japan) at 37°C for 1 h. After spheroplasting efficiency was estimated by measuring OD_600_, spheroplasts were washed with spheroplast wash buffer and lysed with 20 strokes in a Dounce glass homogenizer with a tight pestle (Wheaton Industries, Millville, NJ) in lysis buffer (0.8 mol/L sorbitol; 10 mmol/L triethanolamine, adjusted to pH 7.2 with acetic acid; 1 mmol/L ethylenediaminetetraacetic acid) containing protease inhibitors (1 *μ*g mL^−1^ aprotinin, 1 *μ*g mL^−1^ leupeptin, 1 *μ*g mL^−1^ pepstatin [Peptide institute inc., Osaka, Japan], 2 mmol/L benzamidine [Sigma], and 1 mmol/L phenylmethylsulfonyl fluoride). To prevent nonspecific binding of mRFP-Lact-C2 to SVs, lysis buffer containing 1 mol/L NaCl was added to cell lysates to a final concentration of 0.1 mol/L NaCl. A 700*g* spin for 10 min yielded the pellet (P1) and supernatant (S1) fractions. The S1 fraction was spun at 13,000*g* for 20 min to generate P2 and S2. The S2 fraction was centrifuged at 100,000*g* for 1 h in a 55.2Ti rotor (Beckman Coulter, Fullerton, CA) to generate membrane pellets (P3). For gradient fractionation, an 11 mL 15–30% continuous Nycodenz (Sigma) gradient was created in lysis buffer containing 0.1 mol/L NaCl. The P3 membrane pellets were resuspended in 1 mL of lysis buffer containing 0.1 mol/L NaCl, adjusted to 35% Nycodenz, and loaded on the bottom of the gradient using a 10-cm needle. Gradients were centrifuged in a P40ST rotor (Hitachi, Tokyo, Japan) at 100,000*g* for 16 h, and 0.5-mL fractions were manually collected from the bottom of the tube. Fluorescence intensity of mRFP-Lact-C2 was measured using an FP-6500 spectrofluorometer (Jasco Corp., Tokyo, Japan) at 590 nm (excitation, 530 nm; emission bandwidth, 10 nm; excitation bandwidth, 10 nm; Response, 1.0 sec; Gain, high) and normalized to the OD_600_ equivalent of the spheroplasted cells. For quantitative determination of total phospholipid phosphates in each fraction, lipids were extracted (Bligh and Dyer 1959), and colorimetric assays were performed (Rouser et al. 1970). Fraction densities were determined by measuring refractive indices on a refractometer (PAL-1; ATAGO Co. Ltd., Tokyo, Japan).

### Immunoblot analysis

Immunoblot analysis was performed as described previously (Misu et al. 2003). For SDS-PAGE of Pma1p, samples were heated at 37°C for 15 min before loading. Rabbit anti-RFP (MBL, Nagoya, Japan) and anti-Pma1p (Furuta et al. 2007) polyclonal antibodies were used at 1:2500 and 1:5000 dilution, respectively.

### Microscopic observations

Cells were observed using a Nikon ECLIPSE E800 microscope (Nikon Instec, Tokyo, Japan) equipped with an HB-10103AF superhigh-pressure mercury lamp and a 1.4 numerical aperture 100× Plan Apo oil immersion objective lens (Nikon Instec) with appropriate fluorescence filter sets (Nikon Instec) or differential interference contrast optics. Images were acquired using a cooled digital charge-coupled device camera (C4742-95-12NR; Hamamatsu Photonics, Hamamatsu, Japan) and AQUACOSMOS software (Hamamatsu Photonics). GFP- or mRFP-tagged proteins were observed in living cells, which were grown from early to mid-logarithmic phase, harvested, and resuspended in SD medium. Cells were immediately observed using a GFP bandpass (for GFP) or G2-A (for mRFP) filter set. Treatment with LAT-A (Wako Pure Chemical Industries Ltd.) was performed at 100 *μ*mol/L by addition of a suitable volume of 20 mmol/L stock in dimethyl sulfoxide (DMSO) (Wako Pure Chemical Industries Ltd.) to the medium, as described (Ayscough et al. 1997).

## Results

### PS translocation occurs during vesicle formation from the TGN

**Figure 1:**
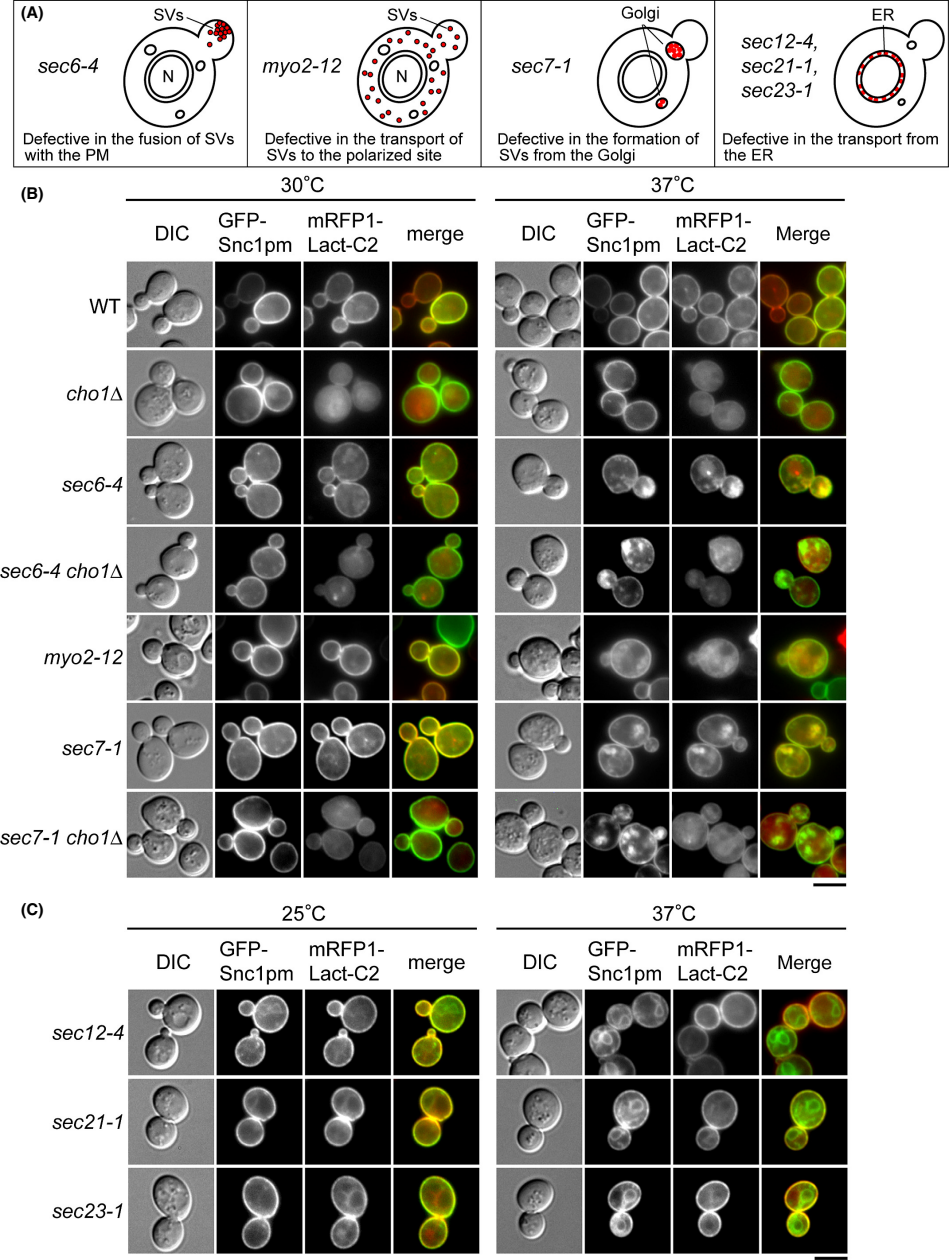
PS is present on the surface of accumulated SVs and the TGN, but not the ER, in secretory mutant cells. (A) Phenotypes of temperature-sensitive *sec* and *myo2* mutants. (B) Localization of mRFP-Lact-C2 to accumulated SVs and TGN membranes in *sec6-4/myo2-12* and *sec7-1* cells, respectively. Cells were incubated for 1 h in SD-Leu medium or SD-Leu medium supplemented with 1 mmol/L ethanolamine (*cho1*Δ mutants) or for 2 h in SD-Leu-Ura medium (*myo2-12* mutant) at 30°C (control) or 37°C. The strains used were wild type (WT) (YKT1843), *cho1*Δ (YKT1845), *sec6-4* (YKT1844), *sec6-4 cho1*Δ (YKT1846), *myo2-12* (YKT1678), *sec7-1* (YKT1857), and *sec7-1 cho1*Δ (YKT1858). These strains, all carrying *mRFP1-Lact-C2* integrated at the *URA3* locus except the *myo2-12* mutant, were transformed with pRS315-GFP-SNC1 pm (pKT1491). The *myo2-12* mutant was cotransformed with pKT1491 and pRS416-mRFP1-Lact-C2 (pKT1755). Bar, 5 *μ*m. (C) mRFP-Lact-C2 did not localize to accumulated ER membranes. Cells were incubated for 1 h in SD-Leu-Ura medium at 25°C (control) or 37°C. The strains used were *sec12-4* (MBY10-11D), *sec21-1* (MBY6-4D), and *sec23-1* (MBY8-20C), all cotransformed with pRS315-GFP-SNC1 pm (pKT1491) and pRS416-mRFP1-Lact-C2 (pKT1755). Bar, 5 *μ*m.

In wild-type cells, mRFP-Lact-C2 was localized to the plasma membrane, but not to any other organelle including the TGN, implying that PS translocation occurs during or after vesicle formation on the TGN (Yeung et al. 2008; Fairn et al. 2011a). To determine at what stage of the secretory pathway PS translocation takes place, we observed mRFP-Lact-C2 in temperature-sensitive secretory pathway mutants (Fig.1A). Vesicle transport through the secretory pathway was monitored using GFP-Snc1p-pm, a plasma membrane-localized mutant v-SNARE containing point mutations that inhibit endocytosis (Lewis et al. 2000).

Sec6p is a subunit of exocyst that is a conserved protein complex required for tethering and fusion of SVs on the plasma membrane (Guo et al. 1999). The *sec6-4* mutation leads to accumulation of post-Golgi plasma membrane-targeted SVs at the restrictive temperature (Novick et al. 1980). When *sec6-4* cells were shifted to 37°C for 1 h, GFP-Snc1p-pm was localized to SVs accumulated in buds (64.9%, *n* = 111 cells), and mRFP-Lact-C2 was colocalized with them (98.5%, *n* = 133 structures) (Fig.1B). This mRFP-Lact-C2 signal disappeared upon deletion of *CHO1*, which encodes a unique PS synthase (Letts et al. 1983). We also examined a mutant that accumulates SVs due to a defect in polarized transport, not that in the exocyst complex. Myo2p, a yeast class V myosin, is required for the actin-based transport of SVs to the polarized site, and loss of the Myo2p function leads to accumulation of SVs throughout cells (Govindan et al. 1995; Schott et al. 1999; Jin et al. 2011). In *myo2-12* cells at 37°C, GFP-Snc1p-pm was diffusely localized to the cytoplasm, and mRFP-Lact-C2 exhibited a similar localization pattern (20.0%, *n* = 120 cells). *SEC7* encodes a guanine nucleotide exchange factor for Arf small GTPases, and the *sec7-1* mutant is defective in formation of SVs from the TGN (Novick et al. 1980; Achstetter et al. 1988). In *sec7-1* cells at 37°C, GFP-Snc1p-pm accumulated in internal membrane structures (76.5%, *n* = 115 cells) identified as enlarged TGN membranes (Novick et al. 1980; Achstetter et al. 1988), with which mRFP-Lact-C2 was also associated (98.2%, *n* = 111 structures). These results are consistent with previous observations (Fairn et al. 2011a). We also examined early secretory pathway mutations (*sec12-4, sec21-1, sec23-1*) that block exit from the ER (Novick et al. 1980). When these mutants were incubated at 37°C, GFP-Snc1p-pm accumulated in the ER (more than 87.6%, *n* > 100 cells), but mRFP-Lact-C2 was not colocalized with these membranes (less than 9.0%, *n* > 100 cells) (Fig.1C).

Taken together, these results suggest that PS translocation occurs during the formation of SVs from the TGN.

### Golgi cisternal maturation may be involved in PS flipping

It is likely that PS flipping occurs concurrently with SV formation from the TGN. However, PS was flipped in the TGN even when SV formation was blocked in the *sec7-1* mutant, suggesting that PS translocation is regulated independently of SV formation. Because mRFP-Lact-C2 did not localize to the ER membrane in mutants defective in ER exit, PS flipping may be specific to the TGN membrane. Therefore, we examined localization of mRFP-Lact-C2 in two mutants defective in membrane traffic in Golgi compartments, *pik1* and *gea1 gea2*.

**Figure 2:**
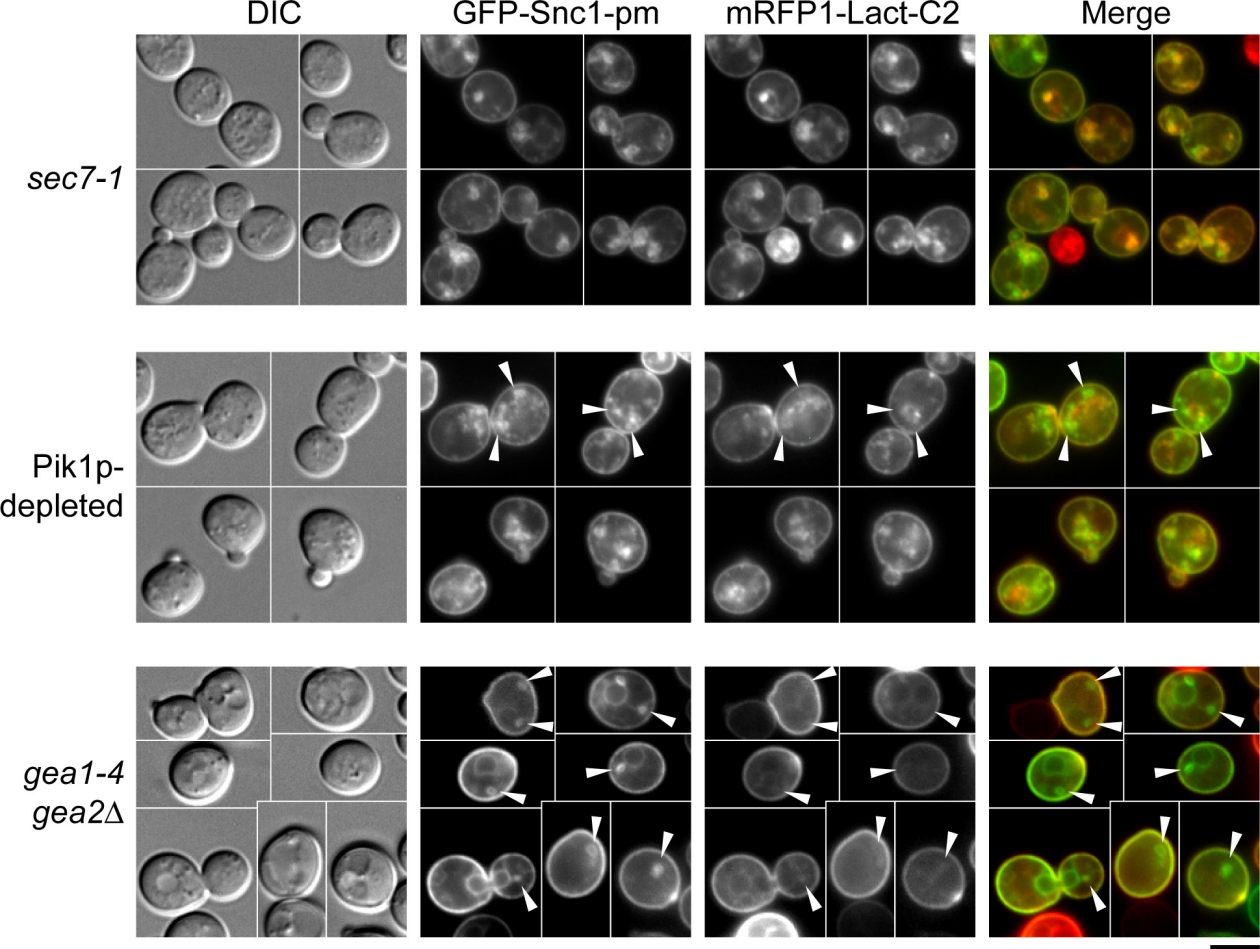
PS flipping is restricted to the TGN membrane. Localization of GFP-Snc1p-pm and mRFP-Lact-C2 was examined in mutants defective in Golgi membrane trafficking. *sec7-1 mRFP1-Lact-C2* (YKT1857) cells carrying pRS315-GFP-SNC1 pm (pKT1491) were incubated in SD-Leu at 37°C for 1 h. To deplete Pik1p, *P*_*GAL1*_*-3HA-PIK1 mRFP1-Lact-C2* (YKT1863) cells carrying pRS315-GFP-SNC1 pm (pKT1491) were incubated in SD-Leu at 30°C for 9 h. *gea1-4 gea2*Δ (CJY062-10-2) cells cotransformed with pRS315-GFP-SNC1 pm (pKT1491) and pRS416-mRFP1-Lact-C2 (pKT1755) were incubated in SD-Leu-Ura at 37°C for 1 h. Arrowheads indicate GFP-Snc1p-pm-positive and mRFP-Lact-C2-negative membrane structures. Bar, 5 *μ*m.

The phosphatidylinositol 4-kinase Pik1p, a binding partner of Sec7p in the late Golgi (Gloor et al. 2010), is required (like Sec7p) for anterograde transport from the TGN to the plasma membrane (Hama et al. 1999; Walch-Solimena and Novick 1999; Audhya et al. 2000). Phosphatidylinositol 4-phosphate (PI(4)P), synthesized at the Golgi by Pik1p, is important for recruitment of a regulator of Rab family small GTPases and clathrin adaptors (Santiago-Tirado and Bretscher 2011). When Pik1p was depleted for 9 h, GFP-Snc1p-pm accumulated throughout the cells (88.8%, *n* = 107 cells). mRFP-Lact-C2 extensively colocalized with GFP-Snc1p-pm, but 11.0% of the cells (*n* = 100 cells) had mRFP-Lact-C2–negative, GFP-Snc1p-pm-containing structures that were hardly detected in *sec7-1* mutant cells (Fig.2, arrowheads). Thus, Pik1p may be involved in the regulation of PS translocation, but it is also possible that these mRFP-Lact-C2–negative membranes are not TGN membranes: instead, given that PI(4)P was shown to be also involved in retrograde Golgi trafficking (Wood et al. 2009), they may be early or medial-Golgi membranes.

Gea1p and Gea2p are Arf1p nucleotide exchange factors that are required for Golgi-to-ER retrograde transport and intra-Golgi transport (Peyroche et al. 2001; Spang et al. 2001). In the *gea1-4 gea2*Δ temperature-sensitive mutant, early and late Golgi enzymes colocalize to a few ring-like structures, suggesting that these structures are derived from early/medial-Golgi membranes (Peyroche et al. 2001). In *gea1-4 gea2*Δ cells, we observed that GFP-Snc1p-pm localized to these structures, as well to the ER (Fig.2, arrowheads; 42.0%, *n* = 200 cells). mRFP-Lact-C2 was only rarely localized to these structures (6.4%, *n* = 173 structures).

Taken together, our results suggest that PS flipping is restricted to TGN membranes. Thus, we conclude that Golgi maturation is a prerequisite for PS translocation.

### Flippase mutations do not reduce PS exposure on accumulated SVs and TGN membranes

**Figure 3:**
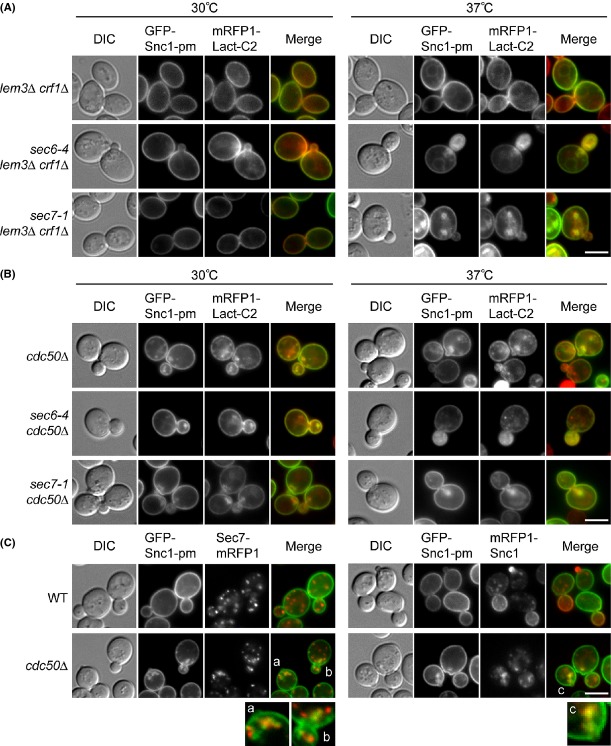
Localization of GFP-Snc1p-pm and mRFP-Lact-C2 in flippase-defective secretory mutant cells. (A) and (B) Localization of GFP-Snc1p-pm and mRFP-Lact-C2 in *lem3*Δ *crf1*Δ and *cdc50*Δ mutants. Cells were incubated in SD-Leu medium at 30°C (control) or 37°C for 1 h. The strains used were *lem3*Δ *crf1*Δ (YKT1847)*, sec6-4 lem3*Δ *crf1*Δ (YKT1848)*, sec7-1 lem3*Δ *crf1*Δ (YKT1860), *cdc50*Δ (YKT1849)*, sec6-4 cdc50*Δ (YKT1850)*,* and *sec7-1 cdc50*Δ (YKT1859), all carrying *URA3::mRFP1-Lact-C2* and pRS315-GFP-SNC1 pm (pKT1491). Bar, 5 *μ*m. (C) Localization of GFP-Snc1p-pm with Sec7p-mRFP or mRFP-Snc1p in the *cdc50*Δ mutant. (Left panel) *SEC7-mRFP1* (YKT1670) and *cdc50*Δ *SEC7-mRFP1* (YKT1149) cells, both carrying pRS416-GFP-SNC1 pm (pKT1444), were incubated in SD-Ura medium at 30°C. (Right panel) Wild-type (YKT38) and *cdc50*Δ (YKT249) cells, both carrying pRS416-GFP-SNC1 pm (pKT1444) and pRS315-mRFP1-SNC1 (pKT1568), were incubated in SD-Leu-Ura medium at 30°C. Regions labeled with small characters are twofold enlarged to compare GFP and mRFP signal patterns. Bar, 5 *μ*m.

We next investigated the contribution of flippases to PS exposure on accumulated SVs and TGN membranes. In the *lem3*Δ *crf1*Δ mutant, both GFP-Snc1p-pm and mRFP-Lact-C2 were exclusively localized to the plasma membrane, as in the wild type (Fig.3A). The *sec6-4 lem3*Δ *crf1*Δ mutant accumulated GFP-Snc1p-pm in buds (66.9%, *n* = 133 cells) that colocalized with mRFP-Lact-C2 (98.6%, *n* = 139 structures) as in *sec6-4* cells. Similarly, as in *sec7-1* cells, the *sec7-1 lem3*Δ *crf1*Δ mutant accumulated GFP-Snc1p-pm membrane structures (62.3%, *n* = 130 cells) that colocalized with mRFP-Lact-C2 (96.8%, *n* = 125 structures). These results suggest that Lem3p-Dnf1/2p and Crf1p-Dnf3p are not essential for PS flipping in the TGN or/and SVs.

We next examined *cdc50*Δ cells and found that this mutant accumulated low levels of GFP-Snc1p-pm, which seemed to be accumulated early endosome/TGN membranes as described below, near polarized sites such as the bud tip or cytokinesis site, both at 30°C (45.8%, *n* = 155 cells) and 37°C (43.5%, *n* = 168 cells) (Fig.3B). mRFP-Lact-C2 was almost entirely colocalized with these structures of GFP-Snc1p-pm, both at 30°C (100%, *n* = 136 structures) and 37°C (96.6%, *n* = 117 structures). Likewise, the *cdc50*Δ mutation did not affect the exposure of PS on the accumulated SVs and TGN membranes (Fig.3B): as in *sec6-4* cells, the *sec6-4 cdc50*Δ mutant accumulated GFP-Snc1p-pm in buds (78.9%, *n* = 133 cells) that colocalized with mRFP-Lact-C2 (97.6%, *n* = 125 structures). Similarly, as in *sec7-1* cells, the *sec7-1 cdc50*Δ mutant accumulated GFP-Snc1p-pm membrane structures (69.7%, *n* = 152 cells) that colocalized with mRFP-Lact-C2 (92.7%, *n* = 137 structures). These results suggest that in the absence of the Cdc50p–Drs2p flippase, PS is exposed on the cytosolic leaflet of the accumulated SVs and TGN membranes.

To characterize the accumulated membranes in the *cdc50*Δ mutant, we compared the localization of GFP-Snc1p-pm with that of Sec7p-mRFP, a TGN marker (Franzusoff et al. 1991). Some GFP-Snc1p-pm structures appeared to overlap with Sec7p-mRFP dots (Fig.3C), but it was difficult to determine whether they were colocalized because these structures were clustered at polarized sites. By contrast, GFP-Snc1p-pm colocalized with mRFP-Snc1p (99.1%, *n* = 113 structures) (Fig.3C), which accumulated due to defects in the endocytic recycling pathway (Saito et al. 2004). These results suggested that GFP-Snc1p-pm accumulated in early endosome/TGN membranes. However, this GFP-Snc1p-pm accumulation did not seem to cause a major defect in the exocytosis pathway, because (1) the *cdc50*Δ cells grew normally at >30°C, and (2) the *cdc50*Δ mutation did not affect recovery of SVs (see below). Thus, exocytic transport is slowed, but not inhibited, in the *cdc50*Δ mutant.

**Figure 4:**
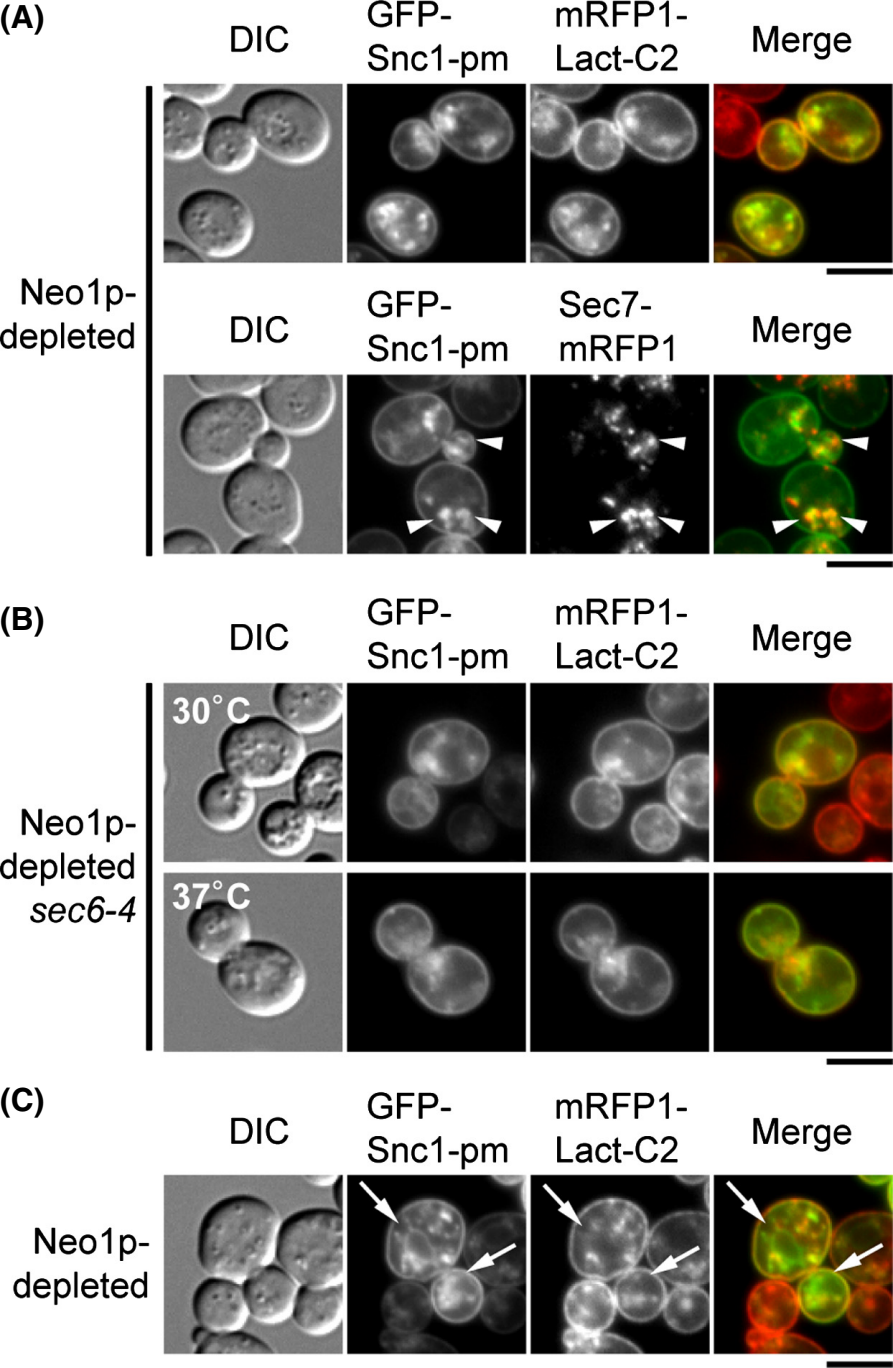
Localization of GFP-Snc1p-pm and mRFP-Lact-C2 in Neo1p-depleted cells. (A) Localization of GFP-Snc1p-pm with mRFP-Lact-C2 or Sec7p-mRFP in Neo1p-depleted cells. To deplete Neo1p, cells were incubated at 30°C for 10.5 h in SD-Ura (for Sec7p-mRFP) or SD-Leu (for mRFP-Lact-C2) medium. The strains used were *P*_*GAL1*_*-3HA-NEO1 SEC7-mRFP1* (YKT1861) carrying pRS416-GFP-SNC1 pm (pKT1444) and *P*_*GAL1*_*-3HA-NEO1 mRFP1-Lact-C2* (YKT1851) carrying pRS315-GFP-SNC1 pm (pKT1491). Bar, 5 *μ*m. (B) Localization of GFP-Snc1p-pm and mRFP-Lact-C2 in the Neo1p-depleted *sec6-4* mutant. Cells were incubated in SD-Leu medium at 30°C for 9.5 h to deplete Neo1p, followed by a shift to 30°C or 37°C for 1 h. The strain used was *sec6-4 P*_*GAL1*_*-3HA-NEO1 mRFP1-Lact-C2* (YKT1852) carrying pRS315-GFP-SNC1 pm (pKT1491). Bar, 5 *μ*m. (C) GFP-Snc1p-pm, but not mRFP-Lact-C2, localized to ER-like membranes in Neo1p-depleted cells. Cells of *P*_*GAL1*_*-3HA-NEO1 mRFP1-Lact-C2* (YKT1851) carrying pRS416-GFP-SNC1 pm (pKT1444) were grown as in (A). Arrows indicate the ER-like membrane structures. Bar, 5 *μ*m.

We next examined the effect of Neo1p depletion on PS flipping. Because *NEO1* is an essential gene (Hua et al. 2002), we depleted Neo1p using the glucose-repressible *GAL1* promoter. When Neo1p was depleted for 10.5 h, GFP-Snc1p-pm accumulated at high levels in intracellular punctate structures (100%, *n* = 113 cells) that significantly overlapped with Sec7p-mRFP (70.8%, *n* = 144 structures) (Fig.4A, arrowheads). These punctate GFP-Snc1p-pm structures colocalized with mRFP-Lact-C2 (90.2%, *n* = 133 structures). Thus, Neo1p may be required for vesicle transport from the TGN, but PS was still flipped at the TGN in the absence of Neo1p. We then reasoned that if Neo1p depletion inhibits vesicle formation from the TGN, it should affect accumulation of SV-associated GFP-Snc1p-pm to the bud in the *sec6-4* mutant. To test this idea, we constructed the *P*_*GAL1*_*-NEO1 sec6-4* strain. Cells were grown at 30°C for 9.5 h in glucose-containing medium to deplete Neo1p, and then shifted to 37°C for 1 h. At 30°C, the Neo1p-depleted *sec6-4* cells accumulated GFP-Snc1p-pm structures (100%, *n* = 104 cells) that colocalized with mRFP-Lact-C2 (86.3%, *n* = 139 punctate structures) (Fig4B), similar to the Neo1p-depleted cells described above. This localization pattern did not significantly change after the shift to 37°C: the GFP-Snc1p-pm structures (99.1%, *n* = 113 cells) were randomly localized, rather than restricted to the bud, and they colocalized with mRFP-Lact-C2 (88.9%, *n* = 144 punctate structures), suggesting that Neo1p depletion may affect SV formation.

In some of the Neo1p-depleted cells, GFP-Snc1p-pm also accumulated in ER-like structures (∼15% at 30°C, Fig.4C, arrows and ∼35% at 37°C, data not shown), consistent with the previous electron microscopic observation that some ER-like membranes accumulated in the *neo1* mutant (Hua and Graham 2003). As in the early *sec* mutants, mRFP-Lact-C2 did not colocalize with these structures.

Taken together, our results suggest that PS exposure on accumulated SVs and TGN membranes is independent of flippases.

### Measurement of mRFP-Lact-C2 fluorescence in isolated SVs

**Figure 5:**
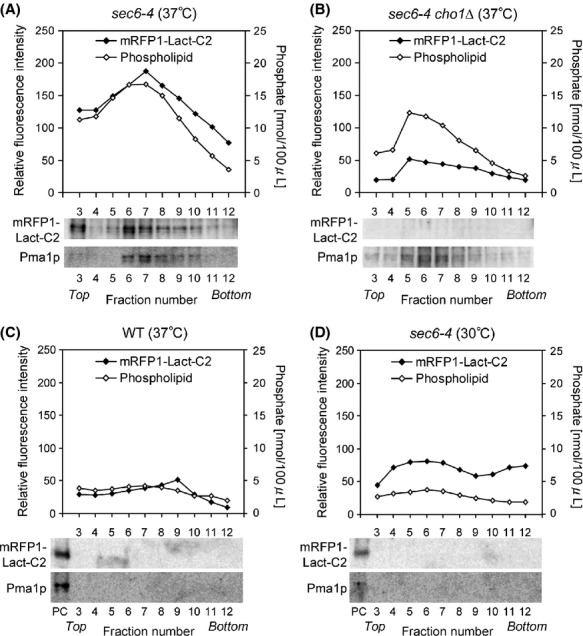
Quantitative analysis of PS on isolated LDSVs by measurement of mRFP-Lact-C2 fluorescence intensity. Cells were grown at 30°C or shifted to 37°C for 2 h. LDSVs were isolated from the cells by subcellular fractionation followed by Nycodenz gradient fractionation. Relative fluorescence intensity of mRFP-Lact-C2 was measured using a spectrofluorometer, and total phospholipid phosphates were determined. Pma1p and mRFP-Lact-C2 were detected by Western blotting using antibodies against Pma1p and RFP, respectively. The SV-enriched fraction from *sec6-4* cells in (A) was loaded as a positive control (PC) for Western blotting in (C) and (D). The strains used were *sec6-4* (YKT1844) (A and D), wild type (WT) (YKT1843) (C), and *sec6-4 cho1*Δ (YKT1846) (B), all carrying *mRFP1-Lact-C2* at the genomic *URA3* locus.

Although mRFP-Lact-C2 appeared to be localized on accumulated SVs or TGN membranes in the absence of flippases, subtle changes in PS concentrations on the surfaces of these vesicles/membranes would not be detectable by fluorescence microscopy. Therefore, to quantitatively analyze PS on the surface of SVs, we isolated SVs from cells expressing mRFP-Lact-C2 in the *sec6-4* background. SVs were purified by subcellular fractionation followed by Nycodenz density gradient centrifugation, based on the procedures described previously (Harsay and Bretscher 1995). The authors who developed these procedures showed that the *sec6-4* mutant accumulates two classes of SVs that differ with regard to both density and cargo proteins: low-density SVs (LDSVs) contain a plasma membrane H^+^-ATPase (Pma1p) activity, whereas high-density SVs (HDSVs) contain the soluble secreted enzymes such as invertase. Isolation of SVs was confirmed by estimating total phospholipid phosphates and Western blotting of Pma1p (Fig.5A). Consistent with a previous report that HDSVs are obtained at much lower yields than LDSVs (Alder-Baerens et al. 2006), we detected only a single peak of phospholipids corresponding to LDSVs (Fig.5A). These phospholipid and Pma1p peaks were not detected in wild-type cells grown at 37°C or *sec6-4* cells grown at 30°C (Fig.5C and D). mRFP-Lact-C2, detected by Western blotting and measurement of mRFP fluorescence intensity using a spectrofluorometer, cofractionated with Pma1p and total phospholipid phosphates (Fig.5A). This mRFP-Lact-C2 peak was not detected in *sec6-4 cho1*Δ cells (Fig.5B), indicating that mRFP-Lact-C2 specifically bound to PS on LDSVs.

Because mRFP-Lact-C2 fluorescence intensity was highly correlated with the amount of total phospholipid phosphates, we concluded that mRFP-Lact-C2 fluorescence could be used as a quantitative measure of PS on LDSVs. HDSVs were not analyzed here, because their amount was not sufficient to give a peak of total phospholipids that is required for calculation of relative PS content.

### Flippase mutations do not reduce PS on isolated LDSVs

**Figure 6:**
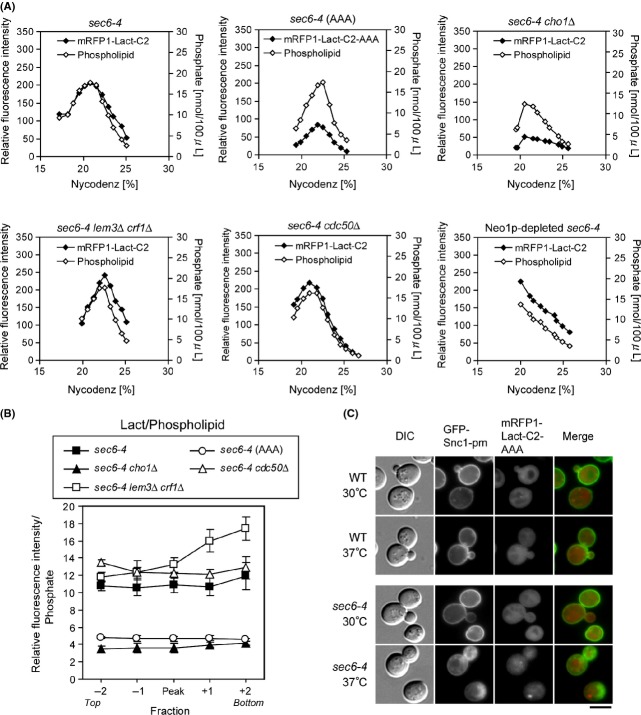
Loss of Lem3p/Crf1p or Cdc50p does not decrease the level of PS in the cytosolic face of LDSVs. (A) Fractionation profile of mRFP-Lact-C2 and total phospholipid phosphates in flippase mutants. Cells were grown in YPDA medium at 30°C and shifted to 37°C for 2 h, whereas *P*_*GAL1*_*-3HA-NEO1 sec6-4* cells were incubated in YPDA medium at 30°C for 8.5 h, followed by a shift to 37°C for 2 h. SVs were isolated and fractionated by Nycodenz density gradient as in Figure5. The strains used were *sec6-4* (YKT1844), *sec6-4 cho1*Δ (YKT1846), *sec6-4 lem3*Δ *crf1*Δ (YKT1848)*, sec6-4 cdc50*Δ (YKT1850), and *sec6-4 P*_*GAL1*_*-3HA-NEO1* (YKT1852), all carrying *mRFP1-Lact-C2* at the genomic *URA3* locus, and *sec6-4* (AAA) (YKT1919) carrying *mRFP1-Lact-C2-AAA* at the genomic *URA3* locus. (B) Lact/Phospholipid in the flippase mutants. Lact/Phospholipid was calculated as the ratio of relative fluorescence intensity of mRFP-Lact-C2 to total phospholipid phosphates in the peak and adjacent four fractions. Data shown are means ± SD of three independent experiments. (C) Localization of GFP-Snc1p-pm and mRFP-Lact-C2-AAA. Cells were incubated in SD-Leu medium at 30°C or 37°C for 1 h. The strains used were *mRFP1-Lact-C2-AAA* (YKT1918) and *sec6-4 mRFP1-Lact-C2-AAA* (YKT1919), both carrying pRS315-GFP-SNC1 pm (pKT1491). Bar, 5 *μ*m.

Using the method described above, we isolated LDSVs from flippase mutants carrying *sec6-4,* and then measured the mRFP-Lact-C2 fluorescence intensity and total phospholipid phosphates (Fig.6A). To quantitatively assess PS content on the surface of LDSVs, we calculated the ratio of mRFP-Lact-C2 fluorescence to total phospholipid phosphates (referred to as Lact/Phospholipid) in the peak and four neighboring fractions (Fig.6B). A mutant version of mRFP-Lact-C2, mRFP-Lact-C2-AAA, which is deficient in PS binding (Yeung et al. 2008), was included as a negative control in addition to *sec6-4 cho1*Δ (Fig.6A, B, and C). Lact/Phospholipid was clearly lower in the *sec6-4 cho1*Δ and *sec6-4 mRFP-Lact-C2-AAA* mutants than in the *sec6-4* mutant.

The *sec6-4 lem3*Δ *crf1*Δ mutant exhibited a peak of mRFP-Lact-C2 comparable to the phospholipid level in *sec6-4*. In denser fractions, Lact/Phospholipid was higher in this mutant than in the *sec6-4* mutant. In the *sec6-4 cdc50*Δ mutant, no marked reduction in the phospholipid level was observed, suggesting that LDSVs were almost normally produced in this mutant. Lact/Phospholipid was slightly higher in this mutant than in the *sec6-4* mutant. These results are essentially consistent with the microscopic observations shown Figure3. On the basis of these findings, we concluded that in the absence of Lem3p–Dnf1/2p and Crf1p–Dnf3p or Cdc50p–Drs2p, PS is flipped during or after SV formation.

Neo1p-depleted *sec6-4* cells, in which Neo1p was depleted for 8.5 h at 30°C followed by a shift to 37°C for 2 h, exhibited a different pattern: total phospholipids were highest in lighter fractions but gradually decreased toward denser fractions. As described for Figure4B, the Neo1p-depleted *sec6-4* cells seem to be deficient in SV formation, because they accumulated GFP-Snc1-pm in aberrant membrane structures, rather than in buds like *sec6-4* cells. Thus, abnormal vesicles or membranes other than SVs might also be obtained by fractionation of the Neo1p-depleted *sec6-4* cells. This abnormal fractionation pattern was not caused by cell death, because about 80% of the cells were alive before cell collection (Fig. S1A). Because Lact/Phospholipid was higher in all fractions in Neo1p-depleted *sec6-4* than in the *sec6-4* single mutant (data not shown), PS may be present in the cytosolic leaflet of these membranes in the absence of Neo1p. As noted above, however, we cannot exclude the possibility that disrupted membranes were also obtained by fractionation of the Neo1p-depleted *sec6-4* cells.

### PS is still exposed on accumulated endosomal/TGN membranes in the simultaneous absence of all known flippases

**Figure 7:**
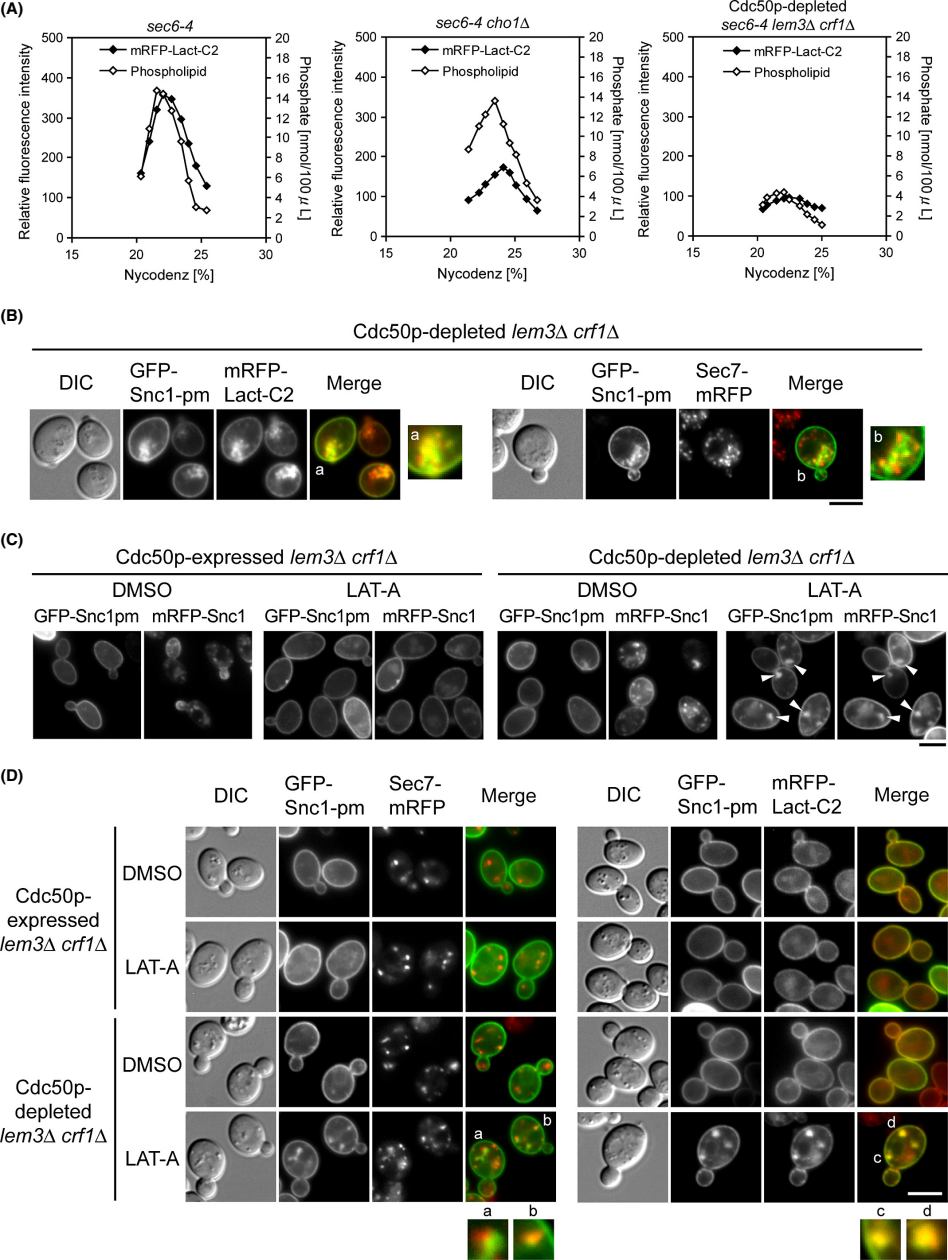
PS is translocated in accumulated endosomal/TGN membranes in the simultaneous absence of multiple flippases. (A) LDSV was not recovered from the Cdc50p-depleted *sec6-4 lem3*Δ *crf1*Δ mutant. The SV fractionation assay (Fig.5) was performed on cells grown in SD medium. Cells were preincubated in SD medium at 30°C for 4 h, followed by an additional 2 h incubation at 37°C. The strains used were *sec6-4 mRFP1-Lact-C2* (YKT1844), *sec6-4 cho1*Δ *mRFP1-Lact-C2* (YKT1846), and *P*_*GAL1*_*-3HA-CDC50 sec6-4 lem3*Δ *crf1*Δ *mRFP1-Lact-C2* (YKT1854). The data shown are representative of four independent experiments. (B) Colocalization of GFP-Snc1p-pm with mRFP-Lact-C2 in the Cdc50p-depleted *lem3*Δ *crf1*Δ mutant. To deplete Cdc50p, cells were incubated at 30°C for 6 h in SD-Leu (for mRFP-Lact-C2) or SD-Ura (for Sec7p-mRFP) medium. Regions indicated with small characters are twofold enlarged to compare GFP and mRFP signal patterns. The strains used were *P*_*GAL1*_*-3HA-CDC50 lem3*Δ *crf1*Δ *mRFP1-Lact-C2* (YKT1853) carrying pRS315-GFP-SNC1 pm (pKT1491) and *P*_*GAL1*_*-3HA-CDC50 lem3*Δ *crf1*Δ *SEC7-mRFP1* (YKT1862) carrying pRS416-GFP-SNC1 pm (pKT1444). Bar, 5 *μ*m. (C) Inhibition of endocytosis caused intracellular accumulation GFP-Snc1p-pm in the partially Cdc50p-depleted *lem3*Δ *crf1*Δ mutant. Cells were incubated in SG-Leu-Ura (Cdc50p-expressing) or SD-Leu-Ura (Cdc50p-depleting) medium at 30°C for 3 h, and then 100 *μ*M LAT-A or DMSO (vehicle control) was added to the medium, followed by further incubation for 1 h. The strain used was *P*_*GAL1*_*-3HA-CDC50 lem3*Δ *crf1*Δ (YKT1103) cotransformed with pRS315-GFP-SNC1 pm (pKT1491) and pRS416-mRFP1-SNC1 (pKT1563). Arrowheads indicate the intracellularly accumulated GFP-Snc1p-pm structures. Bar, 5 *μ*m. (D) Partial colocalization of GFP-Snc1p-pm with Sec7p-mRFP in the LAT-A-treated Cdc50p-depleted *lem3*Δ *crf1*Δ mutant. Cells of the strains in (B) were grown and treated with LAT-A as in (C). Regions labeled with small characters are twofold enlarged to compare GFP and mRFP signal patterns. Bar, 5 *μ*m.

Because it was possible that Lem3p–Dnf1/2p and Crf1p–Dnf3p compensated for the lack of PS flippase activity in the absence of Cdc50p–Drs2p, and *vice versa*, we investigated a mutant lacking all these flippases. Because the *cdc50*Δ mutation is synthetically lethal with the *lem3*Δ *crf1*Δ mutations (Saito et al. 2004), we created the *P*_*GAL1*_*-CDC50 lem3*Δ *crf1*Δ mutant. To quantitatively examine whether the Cdc50p-depleted *lem3*Δ *crf1*Δ mutations would affect the PS level on LDSVs, we tried to perform the SV fractionation assay on the Cdc50p-depleted *sec6-4 lem3*Δ *crf1*Δ cells. However, when the culture was shifted to 37°C for 2 h after Cdc50p was depleted for 4 h at 30°C in YPDA medium, about 50% of the cells died (Fig. S1B). We then attempted the same treatment in synthetic (SD) medium, and found that the cell viability remained high (86.5%) under these conditions (Fig. S1C). Therefore, we performed SV fractionation on the cells grown in SD medium. SVs could be recovered from *sec6-4* cells, as in cells grown in YPDA medium, but the *sec6-4 cho1*Δ mutant exhibited a higher background (4.6-fold) of mRFP-Lact-C2 fluorescence intensity than the cells grown in YPDA medium (Fig.7A), possibly because LDSVs from SD-grown cells contain more PS-independent substance(s) that bind to mRFP-Lact-C2. Nonetheless, Lact/Phospholipid in *sec6-4* cells (20.3 ± 3.7 in the peak fraction of total phospholipid phosphates) was significantly higher than that in *sec6-4 cho1*Δ cells (13.6 ± 1.9). Surprisingly, LDSVs were not recovered from Cdc50p-depleted *sec6-4 lem3*Δ *crf1*Δ cells (Fig.7A), raising the possibility that Cdc50p–Drs2p, Lem3p–Dnf1/2p, and Crf1p–Dnf3p function redundantly in the generation of LDSVs.

We next examined the localization of GFP-Snc1p-pm and mRFP-Lact-C2 in the Cdc50p-depleted *lem3*Δ *crf1*Δ cells. As shown in Figure7B, these cells accumulated GFP-Snc1p-pm near the polarized growth site (50.4%, *n* = 133 cells), and mRFP-Lact-C2 colocalized with those structures (98.1%, *n* = 108 structures). In 84.5% of these cells (*n* = 116 cells), some Sec7p-mRFP dots also clustered to the polarized site, as in *cdc50*Δ cells, and appeared to partially colocalize with the GFP-Snc1p-pm structures. These results suggest that Cdc50p–Drs2p, Lem3p–Dnf1/2p, and Crf1p–Dnf3p may function cooperatively in the transport of GFP-Snc1p-pm from endosomal/TGN membranes, including LDSV formation. Importantly, PS was still exposed on the cytosolic face of these membranes in the absence of these flippases.

We showed previously that these flippases are involved in the endocytic recycling pathway, but not in the secretory pathway from the TGN to the plasma membrane (Furuta et al. 2007). In this study, we isolated and characterized the temperature-sensitive *cdc50-11 lem3*Δ *crf1*Δ mutant, which exhibited normal production of SVs but had defects in endocytic recycling of GFP-Snc1p from early endosome to the TGN. We speculated that a more severe defect in the early endosome-to-TGN pathway in the Cdc50p-depleted *lem3*Δ *crf1*Δ mutant than that in the *cdc50-11 lem3*Δ *crf1*Δ mutant might cause a secondary defect in the secretory pathway. To test this idea, we examined the effect of inhibition of endocytosis on intracellular accumulation of GFP-Snc1p-pm to the TGN. Partial depletion of Cdc50p for 4 h in the *P*_*GAL1*_*-CDC50 lem3*Δ *crf1*Δ mutant resulted in low-level accumulation of mRFP-Snc1p, but not GFP-Snc1p-pm (Fig.7C). However, when endocytic transport was inhibited for 1 h with the actin polymerization inhibitor latrunculin-A (LAT-A) after 3 h depletion of Cdc50p, GFP-Snc1p-pm accumulated in intracellular structures with mRFP-Snc1p in 70.2% of cells (*n* = 114). In 75.9% of cells (*n* = 108 cells), some GFP-Snc1p-pm dots partially colocalized with Sec7p-mRFP structures (Fig.7D). These results are consistent with our notion that inhibition of the endocytic pathway back to the TGN aggravated defects in the Cdc50p-depleted (4 h) *lem3*Δ *crf1*Δ mutant, resulting in a TGN defect. We confirmed that these GFP-Snc1p-pm structures colocalized with mRFP-Lact-C2 (98.3%, *n* = 116 structures) (Fig.7D).

**Figure 8:**
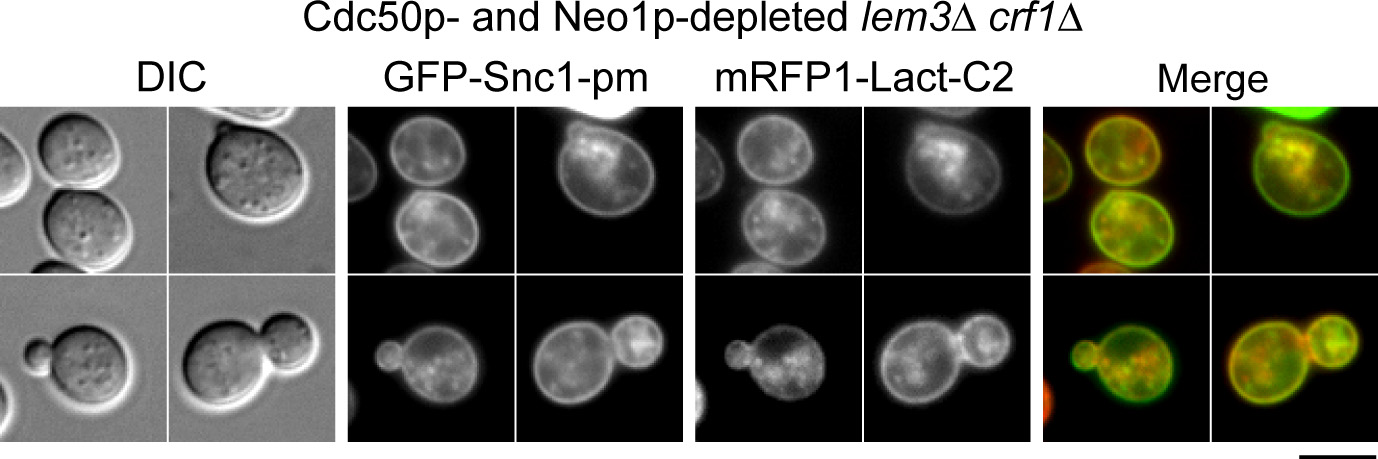
Colocalization of mRFP-Lact-C2 with GFP-Snc1p-pm in a mutant defective in all known flippases. To deplete Cdc50p and Neo1p, cells were incubated in SD-Leu medium at 30°C for 9 h. The strain used was *P*_*GAL1*_*-3HA-CDC50 P*_*GAL1*_*-3HA-NEO1 lem3*Δ *crf1*Δ *mRFP1-Lact-C2* (YKT1856) carrying pRS315-GFP-SNC1 pm (pKT1491). Bar, 5 *μ*m.

Finally, we examined the localizations of GFP-Snc1p-pm and mRFP-Lact-C2 in the Cdc50p- and Neo1p-depleted *lem3*Δ *crf1*Δ mutant. GFP-Snc1p-pm accumulated in almost all cells (93.5%, *n* = 124 cells), and mRFP-Lact-C2 colocalized with these membranes (97.3%, *n* = 111 cells) (Fig.8). We previously showed that mRFP-Lact-C2 also colocalizes with GFP-Snc1p-containing membranes in this mutant (Takeda et al. 2014). We concluded that PS was still flipped in the accumulated endosomal/TGN membranes in the absence of all known flippases.

## Discussion

In this study, we examined the possible involvement of flippases in the generation of plasma membrane PS asymmetry. Taken together with the results of a previous study (Fairn et al. 2011a), our findings suggest that PS has already been flipped in SVs before they fuse with the plasma membrane; that is, PS translocation seems to occur during or after SV formation from the TGN. Phospholipid flippases are candidates for the factors responsible for generating this PS asymmetry. Flippase activity toward PS has been demonstrated for Cdc50p–Drs2p (Natarajan et al. 2004; Alder-Baerens et al. 2006; Zhou and Graham 2009), and Lem3p-Dnf1/2p has also been implicated in PS translocation at the TGN (Hachiro et al. 2013). However, our microscopic observations suggested that none of the flippase mutations significantly changed PS distribution in the membranes we tested, including in the plasma membrane, in LDSVs accumulated in *sec6-4* mutants, or in the enlarged TGN in *sec7-1* mutants. We confirmed these results by quantitatively evaluating, for the first time, the amount of PS on the surface of isolated LDSVs.

We further examined the Cdc50p-depleted *lem3*Δ *crf1*Δ triple mutant, but again, this mutant exhibited localization of mRFP-Lact-C2 to GFP-Snc1p-pm–containing membranes. These membranes partially overlapped with Sec7p-mRFP, and the mutant was defective in LDSV production. On the other hand, we showed previously that the *cdc50-11 lem3*Δ *crf1*Δ mutant accumulated GFP-Snc1p on endosomal membranes due to defects in endocytic recycling (Furuta et al. 2007). The Cdc50p-depleted *lem3*Δ *crf1*Δ mutant accumulated both GFP-Snc1p-pm and mRFP-Snc1p in the same membranes, which probably represented early endosomes and TGN membranes (our unpublished results). Because Snc1p-pm is not endocytosed, it may have been transported to early endosomes from the TGN, where it accumulated. Together, the data led us to the conclusion that PS is exposed on the cytosolic leaflet in the TGN, as well as on early endosomes (Takeda et al. 2014).

We also investigated the possible involvement of Neo1p in PS translocation at the TGN. Neo1p-depleted cells accumulated GFP-Snc1p-pm in the membranes with which Sec7p-mRFP was partially localized, but mRFP-Lact-C2 also localized to these membranes. Finally, we showed that mRFP-Lact-C2 localized to the accumulated endosomal/TGN membranes in the Cdc50p- and Neo1p-depleted *lem3*Δ *crf1*Δ quadruple mutant. In summary, we did not find any evidence that flippases are involved in PS flipping during or after SV formation from the TGN.

**Figure 9:**
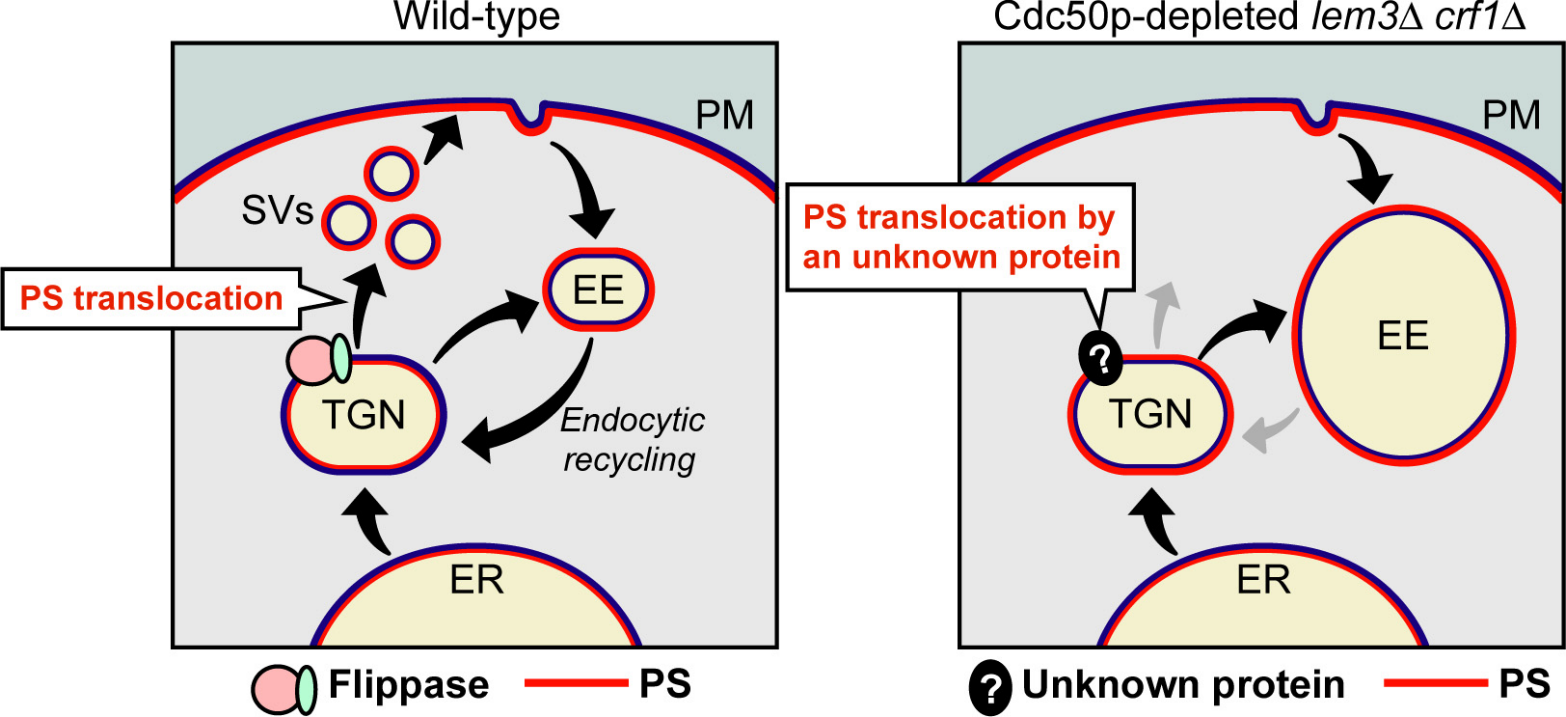
PS distribution and membrane trafficking in Cdc50p-depleted *lem3*Δ *crf1*Δ mutant cells. PS is normally present in the cytosolic leaflet of the plasma membrane and isolated LDSVs, but not in that of the TGN and ER membranes. PS translocation occurs during or after SV formation (Wild type). In Cdc50p-depleted *lem3*Δ *crf1*Δ cells, GFP-Snc1p-pm is accumulated in early endosomal/TGN membranes, which is due to defects in the early endosome-to-TGN pathway and in SV formation from the TGN. PS is exposed in the accumulated endosomal/TGN membranes in the absence of flippases, suggesting that a flippase-like unknown protein is involved in PS translocation at the TGN (Cdc50p-depleted *lem3*Δ *crf1*Δ).

Given the results described above, we hypothesize that a distinct protein with phospholipid translocase activity, in conjunction with flippases, is involved in PS translocation at the TGN (Fig.9). This protein could be another type of ATP-dependent phospholipid translocase; indeed, such an activity has been detected in the plasma membrane by Stevens et al. (2008). Those authors demonstrated that 7-nitrobenz-2-oxa-1,3-diazol-4-yl (NBD)-labeled PS, a fluorescent PS analog commonly used to detect a flippase activity, but not NBD-PC or NBD-PE, translocated across the plasma membrane in an ATP-dependent manner in the absence of Dnf1p, Dnf2p, Dnf3p, and Drs2p. This hypothetical translocase, or a related protein, may also be present in the TGN, like Dnf/Drs2 flippases, which are processed through the endocytic recycling pathway (Saito et al. 2004; Liu et al. 2007). Alternatively, this putative phospholipid translocase could be a scramblase-like protein that bidirectionally exchanges phospholipids across the bilayer in an energy-independent manner (Lhermusier et al. 2011). Because phospholipid translocases are elusive molecules whose activity is difficult to detect from isolated internal membranes such as the Golgi, a combination of genetic, cell biological, and biochemical approaches will be required to identify this protein.

PS flipping appears to occur concomitantly with vesicle formation, but its functional relevance is obscure. mRFP-Lact-C2 localized to isolated LDSVs, but not to TGN membranes in wild-type cells, suggesting that PS translocation occurs during or after vesicle formation. On the other hand, as demonstrated in the *sec7-1* mutant, PS translocation could occur in the TGN membrane independently of SV formation. These results suggest that vesicle formation is not a prerequisite for PS flipping. Consistent with this idea, in mammalian cells PS translocation seems to occur prior to vesicle formation, because mRFP-Lact-C2 is localized to the TGN (Fairn et al. 2011b). Conversely, given that the *cho1*Δ mutant produced LDSVs, PS is not essential for vesicle formation from the TGN. Thus, PS translocation at the TGN seems to be more relevant than vesicle formation to the generation of plasma membrane PS asymmetry.

mRFP-Lact-C2 did not localize to either the ER or *cis-*/medial-Golgi membranes, suggesting that PS flipping was suppressed in these membranes. This could be accomplished by specific localization or/and activation of a putative phospholipid translocase at the TGN. In a previous study, the results of immuno-electron microscopy suggested that PS is present in the luminal leaflet of the ER in mammalian cells (Fairn et al. 2011b). Because PS is synthesized at the cytosolic face of the ER in yeast (Carman and Han 2011), it must be translocated to the luminal leaflet by an unknown mechanism, and then subsequently transported to the TGN and the plasma membrane *via* the secretory pathway. Recently, Maeda et al. (2013) reported another PS transport route to the plasma membrane, in which oxysterol-binding proteins Osh6/7p transport PS from the ER to the plasma membrane *via* a nonvesicular pathway. Thus, PS is efficiently removed from the cytosolic leaflet of the ER by two different mechanisms and transported to the plasma membrane.

## Supporting Information

Figure S1 Cell viability of flippase mutants containing *sec6-4* after flippase depletion and temperature upshift. Cells were incubated in YPGA or YPDA medium at 30°C for 8.5 h (Neo1p depletion) or 4 h (Cdc50p depletion), and then further incubated at 30°C or 37°C for 2 h. Cell viability was determined by PI staining and flow-cytometric analysis. (A) Viability of *PGAL1-3HA-NEO1 sec6-4* cells. The strains used were *PGAL1-3HA-NEO1* (YKT1660) and *sec6-4 PGAL1-3HA-NEO1* (YKT1894). (B) Viability of *sec6-4 PGAL1-3HA-CDC50 lem3*Δ *crf1*Δ cells. The strains used were *PGAL1-3HA-CDC50 lem3*Δ *crf1*Δ (YKT1103) and *sec6-4 PGAL1-3HA-CDC50 lem3*Δ *crf1*Δ (YKT1855). (C) Viability of *sec6-4 PGAL1-3HA-CDC50 lem3*Δ *crf1*Δ cells in SD medium. Viability was determined as in (B), except that cells were grown in SD medium instead of YPDA at 37°C.

## References

[b1] Achstetter T, Franzusoff A, Field C, Schekman R (1988). *SEC7* encodes an unusual, high molecular weight protein required for membrane traffic from the yeast Golgi apparatus. J. Biol. Chem.

[b2] Alder-Baerens N, Lisman Q, Luong L, Pomorski T, Holthuis JC (2006). Loss of P4 ATPases Drs2p and Dnf3p disrupts aminophospholipid transport and asymmetry in yeast post-Golgi secretory vesicles. Mol. Biol. Cell.

[b3] Audhya A, Foti M, Emr SD (2000). Distinct roles for the yeast phosphatidylinositol 4-kinases, Stt4p and Pik1p, in secretion, cell growth, and organelle membrane dynamics. Mol. Biol. Cell.

[b4] Ayscough KR, Stryker J, Pokala N, Sanders M, Crews P, Drubin DG (1997). High rates of actin filament turnover in budding yeast and roles for actin in establishment and maintenance of cell polarity revealed using the actin inhibitor latrunculin-A. J. Cell Biol.

[b5] Bigay J, Antonny B (2012). Curvature, lipid packing, and electrostatics of membrane organelles: defining cellular territories in determining specificity. Dev. Cell.

[b6] Bligh EG, Dyer WJ (1959). A rapid method of total lipid extraction and purification. Can. J. Biochem. Physiol.

[b7] Carman GM, Han GS (2011). Regulation of phospholipid synthesis in the yeast *Saccharomyces cerevisiae*. Annu. Rev. Biochem.

[b8] Chen CY, Ingram MF, Rosal PH, Graham TR (1999). Role for Drs2p, a P-type ATPase and potential aminophospholipid translocase, in yeast late Golgi function. J. Cell Biol.

[b9] Cho KJ, Park JH, Piggott AM, Salim AA, Gorfe AA, Parton RG (2012). Staurosporines disrupt phosphatidylserine trafficking and mislocalize Ras proteins. J. Biol. Chem.

[b10] Daleke DL (2003). Regulation of transbilayer plasma membrane phospholipid asymmetry. J. Lipid Res.

[b11] Daleke DL (2007). Phospholipid flippases. J. Biol. Chem.

[b12] Das A, Slaughter BD, Unruh JR, Bradford WD, Alexander R, Rubinstein B (2012). Flippase-mediated phospholipid asymmetry promotes fast Cdc42 recycling in dynamic maintenance of cell polarity. Nat. Cell Biol.

[b13] Elble R (1992). A simple and efficient procedure for transformation of yeasts. Biotechniques.

[b14] Fairn GD, Hermansson M, Somerharju P, Grinstein S (2011a). Phosphatidylserine is polarized and required for proper Cdc42 localization and for development of cell polarity. Nat. Cell Biol.

[b15] Fairn GD, Schieber NL, Ariotti N, Murphy S, Kuerschner L, Webb RI (2011b). High-resolution mapping reveals topologically distinct cellular pools of phosphatidylserine. J. Cell Biol.

[b16] Franzusoff A, Redding K, Crosby J, Fuller RS, Schekman R (1991). Localization of components involved in protein transport and processing through the yeast Golgi apparatus. J. Cell Biol.

[b17] Furuta N, Fujimura-Kamada K, Saito K, Yamamoto T, Tanaka K (2007). Endocytic recycling in yeast is regulated by putative phospholipid translocases and the Ypt31p/32p-Rcy1p pathway. Mol. Biol. Cell.

[b18] Gall WE, Geething NC, Hua Z, Ingram MF, Liu K, Chen SI (2002). Drs2p-dependent formation of exocytic clathrin-coated vesicles in vivo. Curr. Biol.

[b19] Gietz RD, Woods RA (2002). Transformation of yeast by lithium acetate/single-stranded carrier DNA/polyethylene glycol method. Methods Enzymol.

[b20] Gloor Y, Schöne M, Habermann B, Ercan E, Beck M, Weselek G (2010). Interaction between Sec7p and Pik1p: the first clue for the regulation of a coincidence detection signal. Eur. J. Cell Biol.

[b21] Govindan B, Bowser R, Novick P (1995). The role of Myo2, a yeast class V myosin, in vesicular transport. J. Cell Biol.

[b22] Guo W, Roth D, Walch-Solimena C, Novick P (1999). The exocyst is an effector for Sec4p, targeting secretory vesicles to sites of exocytosis. EMBO J.

[b23] Guthrie C, Fink GR (1991). Guide to yeast genetics and molecular biology.

[b24] Hachiro T, Yamamoto T, Nakano K, Tanaka K (2013). Phospholipid flippases Lem3p-Dnf1p and Lem3p-Dnf2p are involved in the sorting of the tryptophan permease Tat2p in yeast. J. Biol. Chem.

[b25] Hama H, Schnieders EA, Thorner J, Takemoto JY, DeWald DB (1999). Direct involvement of phosphatidylinositol 4-phosphate in secretion in the yeast *Saccharomyces cerevisiae*. J. Biol. Chem.

[b26] Hanamatsu H, Fujimura-Kamada K, Yamamoto T, Furuta N, Tanaka K (2014). Interaction of the phospholipid flippase Drs2p with the F-box protein Rcy1p plays an important role in early endosome to *trans*-Golgi network vesicle transport in yeast. J. Biochem.

[b27] Harsay E, Bretscher A (1995). Parallel secretory pathways to the cell surface in yeast. J. Cell Biol.

[b28] Hua Z, Graham TR (2003). Requirement for Neo1p in retrograde transport from the Golgi complex to the endoplasmic reticulum. Mol. Biol. Cell.

[b29] Hua Z, Fatheddin P, Graham TR (2002). An essential subfamily of Drs2p-related P-type ATPases is required for protein trafficking between Golgi complex and endosomal/vacuolar system. Mol. Biol. Cell.

[b30] Jin Y, Sultana A, Gandhi P, Franklin E, Hamamoto S, Khan AR (2011). Myosin V transports secretory vesicles via a Rab GTPase cascade and interaction with the exocyst complex. Dev. Cell.

[b31] Kato U, Emoto K, Fredriksson C, Nakamura H, Ohta A, Kobayashi T (2002). A novel membrane protein, Ros3p, is required for phospholipid translocation across the plasma membrane in *Saccharomyces cerevisiae*. J. Biol. Chem.

[b32] Kay JG, Koivusalo M, Ma X, Wohland T, Grinstein S (2012). Phosphatidylserine dynamics in cellular membranes. Mol. Biol. Cell.

[b33] Lenoir G, Williamson P, Holthuis JC (2007). On the origin of lipid asymmetry: the flip side of ion transport. Curr. Opin. Chem. Biol.

[b34] Lenoir G, Williamson P, Puts CF, Holthuis JC (2009). Cdc50p plays a vital role in the ATPase reaction cycle of the putative aminophospholipid transporter Drs2p. J. Biol. Chem.

[b35] Letts VA, Klig LS, Bae-Lee M, Carman GM, Henry SA (1983). Isolation of the yeast structural gene for the membrane-associated enzyme phosphatidylserine synthase. Proc. Natl. Acad. Sci. USA.

[b36] Lewis MJ, Nichols BJ, Prescianotto-Baschong C, Riezman H, Pelham HR (2000). Specific retrieval of the exocytic SNARE Snc1p from early yeast endosomes. Mol. Biol. Cell.

[b37] Lhermusier T, Chap H, Payrastre B (2011). Platelet membrane phospholipid asymmetry: from the characterization of a scramblase activity to the identification of an essential protein mutated in Scott syndrome. J. Thromb. Haemost.

[b38] Liu K, Hua Z, Nepute JA, Graham TR (2007). Yeast P4-ATPases Drs2p and Dnf1p are essential cargos of the NPFXD/Sla1p endocytic pathway. Mol. Biol. Cell.

[b39] Liu K, Surendhran K, Nothwehr SF, Graham TR (2008). P4-ATPase requirement for AP-1/clathrin function in protein transport from the *trans*-Golgi network and early endosomes. Mol. Biol. Cell.

[b40] Longtine MS, McKenzie A, Demarini DJ, Shah NG, Wach A, Brachat A (1998). Additional modules for versatile and economical PCR-based gene deletion and modification in *Saccharomyces cerevisiae*. Yeast.

[b41] Maeda K, Anand K, Chiapparino A, Kumar A, Poletto M, Kaksonen M (2013). Interactome map uncovers phosphatidylserine transport by oxysterol-binding proteins. Nature.

[b42] Misu K, Fujimura-Kamada K, Ueda T, Nakano A, Katoh H, Tanaka K (2003). Cdc50p, a conserved endosomal membrane protein, controls polarized growth in *Saccharomyces cerevisiae*. Mol. Biol. Cell.

[b43] Natarajan P, Wang J, Hua Z, Graham TR (2004). Drs2p-coupled aminophospholipid translocase activity in yeast Golgi membranes and relationship to in vivo function. Proc. Natl. Acad. Sci. USA.

[b44] Novick P, Field C, Schekman R (1980). Identification of 23 complementation groups required for post-translational events in the yeast secretory pathway. Cell.

[b45] Peyroche A, Courbeyrette R, Rambourg A, Jackson CL (2001). The ARF exchange factors Gea1p and Gea2p regulate Golgi structure and function in yeast. J. Cell Sci.

[b46] Pomorski T, Lombardi R, Riezman H, Devaux PF, van Meer G, Holthuis JC (2003). Drs2p-related P-type ATPases Dnf1p and Dnf2p are required for phospholipid translocation across the yeast plasma membrane and serve a role in endocytosis. Mol. Biol. Cell.

[b47] Puts CF, Panatala R, Hennrich H, Tsareva A, Williamson P, Holthuis JC (2012). Mapping functional interactions in a heterodimeric phospholipid pump. J. Biol. Chem.

[b48] Ravichandran KS, Lorenz U (2007). Engulfment of apoptotic cells: signals for a good meal. Nat. Rev. Immunol.

[b49] Rose MD, Winston F, Hieter P (1990). Methods in yeast genetics: a laboratory course manual.

[b50] Rouser G, Fleischer S, Yamamoto A (1970). Two dimensional thin layer chromatographic separation of polar lipids and determination of phospholipids by phosphorus analysis of spots. Lipids.

[b51] Saito K, Fujimura-Kamada K, Furuta N, Kato U, Umeda M, Tanaka K (2004). Cdc50p, a protein required for polarized growth, associates with the Drs2p P-type ATPase implicated in phospholipid translocation in *Saccharomyces cerevisiae*. Mol. Biol. Cell.

[b52] Saito K, Fujimura-Kamada K, Hanamatsu H, Kato U, Umeda M, Kozminski KG (2007). Transbilayer phospholipid flipping regulates Cdc42p signaling during polarized cell growth via Rga GTPase-activating proteins. Dev. Cell.

[b53] Santiago-Tirado FH, Bretscher A (2011). Membrane-trafficking sorting hubs: cooperation between PI4P and small GTPases at the *trans*-Golgi network. Trends Cell Biol.

[b54] Schott D, Ho J, Pruyne D, Bretscher A (1999). The COOH-terminal domain of Myo2p, a yeast myosin V, has a direct role in secretory vesicle targeting. J. Cell Biol.

[b55] Sebastian TT, Baldridge RD, Xu P, Graham TR (2012). Phospholipid flippases: building asymmetric membranes and transport vesicles. Biochim. Biophys. Acta.

[b56] Spang A, Herrmann JM, Hamamoto S, Schekman R (2001). The ADP ribosylation factor-nucleotide exchange factors Gea1p and Gea2p have overlapping, but not redundant functions in retrograde transport from the Golgi to the endoplasmic reticulum. Mol. Biol. Cell.

[b57] Stevens HC, Malone L, Nichols JW (2008). The putative aminophospholipid translocases, *DNF1* and *DNF2*, are not required for 7-nitrobenz-2-oxa-1,3-diazol-4-yl-phosphatidylserine flip across the plasma membrane of *Saccharomyces cerevisiae*. J. Biol. Chem.

[b58] Takahashi Y, Fujimura-Kamada K, Kondo S, Tanaka K (2011). Isolation and characterization of novel mutations in *CDC50*, the non-catalytic subunit of the Drs2p phospholipid flippase. J. Biochem.

[b59] Takeda M, Yamagami K, Tanaka K (2014). Role of phosphatidylserine in phospholipid flippase-mediated vesicle transport in *Saccharomyces cerevisiae*. Eukaryot. Cell.

[b60] Tanaka K, Fujimura-Kamada K, Yamamoto T (2011). Functions of phospholipid flippases. J. Biochem.

[b61] Walch-Solimena C, Novick P (1999). The yeast phosphatidylinositol-4-OH kinase Pik1 regulates secretion at the Golgi. Nat. Cell Biol.

[b62] Wicky S, Schwarz H, Singer-Krüger B (2004). Molecular interactions of yeast Neo1p, an essential member of the Drs2 family of aminophospholipid translocases, and its role in membrane trafficking within the endomembrane system. Mol. Cell. Biol.

[b63] Wood CS, Schmitz KR, Bessman NJ, Setty TG, Ferguson KM, Burd CG (2009). PtdIns4P recognition by Vps74/GOLPH3 links PtdIns 4-kinase signaling to retrograde Golgi trafficking. J. Cell Biol.

[b64] Yamamoto T, Mochida J, Kadota J, Takeda M, Bi E, Tanaka K (2010). Initial polarized bud growth by endocytic recycling in the absence of actin cable–dependent vesicle transport in yeast. Mol. Biol. Cell.

[b65] Yeung T, Gilbert GE, Shi J, Silvius J, Kapus A, Grinstein S (2008). Membrane phosphatidylserine regulates surface charge and protein localization. Science.

[b66] Zhou X, Graham TR (2009). Reconstitution of phospholipid translocase activity with purified Drs2p, a type-IV P-type ATPase from budding yeast. Proc. Natl. Acad. Sci. USA.

