# Supplementary material for: Asymmetric distribution of phosphatidylserine is generated in the absence of phospholipid flippases in Saccharomyces cerevisiae
Source: Microbiologyopen. 2014 Sep 13;3(5):803–21. doi: 10.1002/mbo3.211 (PMC4234269; doi:10.1002/mbo3.211)
Supplement: Figure S1 — Cell viability of flippase mutants containing sec6-4 after flippase depletion and temperature upshift. Cells were incubated in YPGA or YPDA medium at 30°C for 8.5 h (Neo1p depletion) or 4 h (Cdc50p depletion), and then further incubated at 30°C or 37°C for 2 h. Cell viability was determined by PI staining and flow-cytometric analysis. (A) Viability of PGAL1-3HA-NEO1 sec6-4 cells. The strains used were PGAL1-3HA-NEO1 (YKT1660) and sec6-4 PGAL1-3HA-NEO1 (YKT1894). (B) Viability of sec6-4 PGAL1-3HA-CDC50 lem3Δ crf1Δ cells. The strains used were PGAL1-3HA-CDC50 lem3Δ crf1Δ (YKT1103) and sec6-4 PGAL1-3HA-CDC50 lem3Δ crf1Δ (YKT1855). (C) Viability of sec6-4 PGAL1-3HA-CDC50 lem3Δ crf1Δ cells in SD medium. Viability was determined as in (B), except that cells were grown in SD medium instead of YPDA at 37°C. [file mbo30003-0803-sd1.pdf]

Figure S1

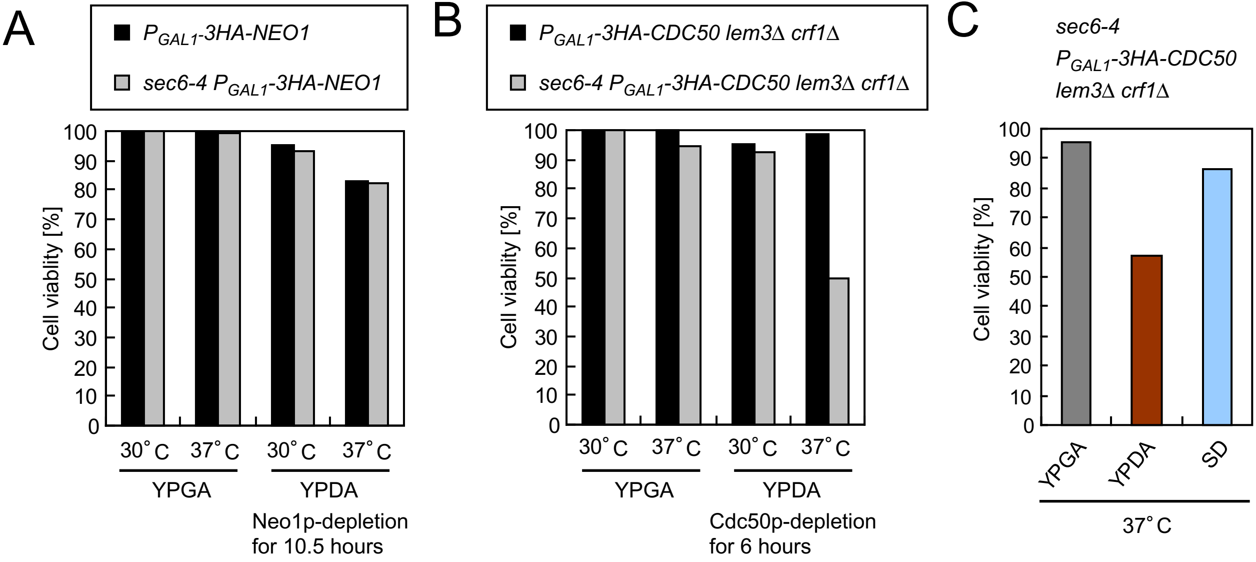
A
P_{GAL1}-3HA-NEO1
sec6-4 P_{GAL1}-3HA-NEO1
Cell viability [%]
100
90
80
70
60
50
40
30
20
10
0
30° C
37° C
30° C
37° C
YPGA
YPDA
Neo1p-depletion
for 10.5 hours
B
P_{GAL1}-3HA-CDC50 lem3Δ crf1Δ
sec6-4 P_{GAL1}-3HA-CDC50 lem3Δ crf1Δ
Cell viability [%]
30° C
37° C
30° C
37° C
YPGA
YPDA
Cdc50p-depletion
for 6 hours
C
sec6-4
P_{GAL1}-3HA-CDC50
lem3Δ crf1Δ
Cell viability [%]
YPGA
YPDA
SD
37° C

Supplemental Figure S1. Cell viability of flippase mutants containing *sec6-4* after flippase depletion and temperature up-shift. Cells were incubated in YPGA or YPDA medium at 30°C for 8.5 h (Neo1p depletion) or 4 h (Cdc50p depletion), and then further incubated at 30°C or 37°C for 2 h. Cell viability was determined by PI staining and flow-cytometric analysis. (A) Viability of P_{GAL1}*-3HA-NEO1 sec6-4* cells. The strains used were P_{GAL1}*-3HA-NEO1* (YKT1660) and *sec6-4* P_{GAL1}*-3HA-NEO1* (YKT1894). (B) Viability of *sec6-4* P_{GAL1}*-3HA-CDC50 lem3*Δ *crf1*Δ cells. The strains used were P_{GAL1}*-3HA-CDC50 lem3*Δ *crf1*Δ (YKT1103) and *sec6-4* P_{GAL1}*-3HA-CDC50 lem3*Δ *crf1*Δ (YKT1855). (C) Viability of *sec6-4* P_{GAL1}*-3HA-CDC50 lem3*Δ *crf1*Δ cells in SD medium. Viability was determined as in (B), except that cells were grown in SD medium instead of YPDA at 37°C.
